# Architecture-driven enhancement of tribological performance, surface integrity, and hardness in magnesium-carbon fiber hybrid sandwich composites for key aerospace applications

**DOI:** 10.1038/s41598-026-54926-x

**Published:** 2026-06-03

**Authors:** M. E. Annadorai, M. Ramakrishna

**Affiliations:** Department of Mechanical Engineering, VFSTR (Deemed to be University), Vadlamudi, Guntur, AP India

**Keywords:** Magnesium-carbon fiber composites, Sandwich composites, Fiber architecture, Dry sliding wear, Wear resistance, Coefficient of friction, Surface roughness, Microhardness, Aerospace materials, Engineering, Materials science

## Abstract

Magnesium-carbon fiber (Mg-CF) sandwich composites are promising lightweight materials for aerospace applications; however, their tribological performance and surface durability under sliding conditions remain insufficiently understood, particularly regarding the influence of fiber architecture. This study addresses this gap by investigating the wear behaviour, surface roughness evolution, and hardness characteristics of Mg-CF sandwich composites with different fiber orientations. The composites were fabricated using filament drum winding, hand lay-up, and compression moulding, incorporating unidirectional ([0°], [45°], [90°]), bidirectional ([0°/90°], [45°/−45°]), and multidirectional ([0°/90°/45°/−45°]) stacking configurations. Tribological performance was evaluated using a pin-on-disc test under dry sliding conditions (20 N load, 300 rpm, and 848 m sliding distance) to determine the specific wear rate and coefficient of friction. Surface roughness and Vickers microhardness were analysed to support tribological performance, while scanning electron microscopy was used to identify dominant wear mechanisms. The results demonstrate that fiber architecture plays a decisive role in wear resistance, with the multidirectional laminate (Mg/CF[0°/90°/45°/−45°]_2_/Mg) exhibiting the lowest specific wear rate (0.86 × 10⁻³ mm³/N·m), corresponding to an improvement of approximately 44% compared to the unidirectional configuration. The reduced wear is supported by a lower coefficient of friction (0.145), minimal surface roughness increment (0.42 μm), and enhanced cross-sectional hardness (47.27 HV), indicating improved load distribution and interfacial stability. SEM observations revealed predominantly abrasive wear with reduced damage in multidirectional configurations. Overall, the study establishes a clear structure-tribology relationship, demonstrating that optimized fiber architectures significantly enhance wear resistance and surface integrity in Mg-CF sandwich composites.

## Introduction

In aerospace engineering, the demand for lightweight, high-performance materials has driven extensive research into advanced composite structures capable of delivering superior mechanical performance and surface durability. Among various structural concepts, sandwich composites have gained significant attention due to their high stiffness-to-weight ratio, excellent specific strength, and enhanced thermal and impact resistance. These attributes make them highly suitable for aircraft, spacecraft, and high-performance vehicle structures where strength and weight efficiency are critical^1,2^.

Magnesium-based composites have emerged as promising alternatives to conventional aluminium and titanium alloys owing to their low density, high specific stiffness, and excellent damping capacity. Among them, AZ-series magnesium alloys offer a favourable combination of strength, formability, and manufacturability, making them attractive for lightweight structural applications^3,4^. However, their broader application is limited by inherent brittleness, relatively poor wear resistance, and susceptibility to surface damage. Reinforcement with high-performance fibres such as carbon fibre provides an effective strategy to enhance both structural integrity and surface performance^5^.

Carbon fibres exhibit exceptional strength, high modulus, thermal stability, and corrosion resistance, making them suitable for advanced composite systems^6^. When combined with magnesium face sheets in a sandwich configuration, carbon fiber-reinforced polymer (CFRP) cores facilitate efficient load transfer, improved damage tolerance, and enhanced durability. In such systems, fibre orientation and stacking sequence play a critical role in governing stress distribution, interfacial stability, and overall performance^7,8^. While previous studies have primarily focused on mechanical and thermal behaviour, investigations on tribological response and surface degradation remain limited.

In practical aerospace applications, sandwich components are frequently subjected to sliding contact, edge wear, fretting, and localised indentation during assembly and service, leading to progressive surface degradation that can significantly affect frictional behaviour, wear resistance, and long-term reliability^9,10^. Surface roughness evolution during sliding is particularly critical, as it influences real contact area, debris formation, and wear mechanisms. Similarly, hardness is a key indicator of resistance to localised plastic deformation and is closely linked to wear performance^11,12^. Despite increasing research on magnesium-based hybrid systems, a comprehensive understanding of their tribological behaviour in sandwich configurations remains limited. Existing studies predominantly focus on bulk properties, with comparatively less attention to surface degradation mechanisms, interfacial behaviour, and thickness-dependent hardness distribution. In particular, the influence of fibre orientation and stacking sequence on cross-sectional wear behaviour has not been systematically explored.

This study uniquely investigates the influence of fiber orientation-dependent load transfer mechanisms on the cross-sectional tribological behaviour of magnesium-carbon fiber sandwich composites. Unlike conventional surface wear studies, the present work focuses on cross-sectional architecture-driven wear response, enabling direct assessment of interfacial load sharing and subsurface damage evolution. This approach provides new insights into orientation-controlled wear mechanisms, which remain insufficiently explored in existing Mg-CFRP tribology studies. In contrast to conventional surface wear testing, the cross-sectional wear evaluation adopted in this study enables direct assessment of the combined tribological response of the magnesium face sheets, CFRP core, and their interfacial regions^13^. This approach provides a more realistic representation of load transfer and subsurface damage evolution in layered and hybrid composite systems. Similar methodologies have been employed in the tribological analysis of multi-material and layered composites to capture interfacial wear mechanisms and material interaction effects more effectively than surface-only testing^14,15^. Therefore, the present approach offers improved insight into architecture-dependent wear behaviour in sandwich structures.

## Literature review

Previous studies on magnesium-based hybrid and sandwich composites have highlighted the dominant role of reinforcement architecture and interfacial integrity in governing tribological and surface performance^16^. Carbon fiber-reinforced magnesium systems generally exhibit lower friction coefficients and reduced wear volume compared to monolithic alloys, primarily due to improved load sharing, fiber bridging, and enhanced resistance to crack initiation and propagation at the sliding interface^17,18^. Zhang et al. reported that carbon fibers promote smoother wear tracks and improved surface integrity by stabilising debris formation and reducing interfacial shear stress under dry sliding conditions^19^. Similarly, investigations on AZ-series magnesium alloys (AZ31, AZ61, and AZ91) reinforced with ceramic particles such as SiC, Al₂O₃, TiC, and B₄C demonstrate significant reductions in wear rate and coefficient of friction due to increased hardness and suppression of subsurface plastic deformation^20,21^.

However, the reported tribological performance varies depending on reinforcement type and architecture^22^. While particle-reinforced systems enhance hardness and wear resistance, they may also introduce brittleness and interfacial stress concentrations under cyclic or sliding loads. In contrast, continuous fiber-reinforced systems provide improved load transfer and damage tolerance, but their performance is highly sensitive to fibre orientation, interfacial bonding, and processing quality^23,24^. These differences indicate that the tribological behaviour of magnesium-based composites cannot be generalised and must be evaluated considering architecture-specific effects. Laminate architecture and fiber orientation have been identified as critical factors influencing wear and friction behaviour. Unidirectional laminates exhibit pronounced anisotropic wear and accelerated surface damage when sliding occurs transverse to the fiber direction. In contrast, bidirectional and multidirectional configurations enable improved stress redistribution, reduced fiber pull-out, and enhanced resistance to delamination, resulting in more stable friction behaviour and lower wear rates^25–27^.

Nevertheless, conflicting observations exist, as some studies report that multidirectional stacking may introduce interfacial discontinuities and localised stress concentrations depending on fabrication quality and fibre alignment, thereby affecting wear stability^28^. Surface roughness evolution further reflects the role of interfacial integrity in tribological performance. Magnesium-based composites with weak bonding or poor reinforcement distribution tend to develop severe ploughing grooves, adhesive transfer layers, and microcracks, leading to significant roughness increase after wear. Conversely, systems with strong interfacial bonding exhibit smoother wear tracks and reduced roughness, indicating improved debris stability and surface integrity^29–31^. Hardness has also been consistently correlated with wear behaviour, where increased microhardness reduces wear rate and enhances resistance to localised plastic deformation^32–34^. Nevertheless, most reported hardness measurements in sandwich composites are limited to surface or face sheet regions, with limited emphasis on cross-sectional hardness distribution capturing fibre orientation and interfacial effects across the thickness.

Despite these advancements, important limitations remain. Most existing studies focus on monolithic or particle-reinforced magnesium systems, with comparatively fewer investigations on hybrid sandwich structures incorporating continuous carbon fibre reinforcement. Furthermore, prior research predominantly emphasises surface-level tribological behaviour, with limited attention to the coupled response of face sheets, core, and interfaces under sliding conditions. In particular, the quantitative influence of fibre orientation on (i) cross-sectional wear rate under multi-material contact, (ii) thickness-dependent hardness distribution across the sandwich structure, and (iii) surface roughness evolution during sliding has not been systematically investigated. The absence of such integrated analysis limits the understanding of architecture-dependent tribological mechanisms in Mg-CF sandwich composites. Accordingly, the present study addresses these specific gaps by evaluating orientation-dependent wear behaviour, surface degradation, and cross-sectional hardness characteristics under controlled dry sliding conditions, with the objective of establishing clear structure-property relationships.

## Materials & methodology

### Materials used

AZ31 magnesium alloy sheets were selected as face materials due to their low density, good formability, and suitability for lightweight aerospace structures. The AZ31 magnesium alloy used in this study exhibits a tensile strength of approximately 172.88 MPa and an elastic modulus of 45.7 GPa, which is consistent with reported values for AZ31 alloys (40–45 GPa), confirming the reliability of the measurements. The observed tensile strength falls within the expected range for rolled AZ31 sheets and is influenced by crystallographic texture and deformation direction. The alloy also possesses a density of 1.77 g/cm³ and a thermal conductivity of 96 W/m·K, making it suitable for lightweight structural and thermal applications. High strength 3k carbon fiber tows were used as reinforcement in the core. The carbon fiber roving (3k yarn), consisting of continuous filaments, exhibits a tensile strength of approximately 3429.2 MPa, an elastic modulus of 230 GPa, and a density of 1.79 g/cm³, enabling efficient load transfer and high stiffness in the composite structure. The matrix system consisted of LY556 epoxy resin and Aradur 5200 hardener. The resin and hardener were mixed in a weight ratio of 100:23, as recommended by the manufacturer, to ensure proper curing and optimal mechanical performance^35–37^. The material properties listed in Table 1 are obtained from a combination of experimental measurements and standard literature values for AZ31 magnesium alloy and carbon fiber, as referenced accordingly. The properties listed in Table 2 are obtained from the manufacturer’s technical datasheet for the epoxy resin and hardener used in this study.

**Table 1:** Properties of the AZ31 magnesium alloy sheet and carbon fiber yarn roving^38–40^.

Material name	Form	Function	Tensile strength (MPa)	Elastic modulus (GPa)	Density (g/cm³)	Thermal conductivity (W/m-K)	Maximum Temp (°C)
AZ31 Mg Alloy	Rolled Sheet	Face Sheet	172.88	45.7	1.77	96.0	605–650
Carbon Fiber	Continuous Tow	Core	3429.2	230	1.79	64–120	2000–3000 (non-inert atm)

**Table 2:** Specifications of epoxy resin system and curing parameters used in composite fabrication^39^.

Material name	Form	Function	Density	Viscosity	Gel time	Curing temperature	Pot life
Epoxy LY556	Liquid gel	Resin	1.15–1.20 g/cm³ at 25 °C	10,000–12,500 mPa·s at 25 °C	80 °C for 2 h	120 °C for 4 h	-
Aradur 5200	Liquid	Hardner	1.20 g/cm³ at 25 °C	500-1,000 mPa·s at 25 °C	-	-	2–4 h

### Fabrication of sandwich composites

**Fig. 1:**
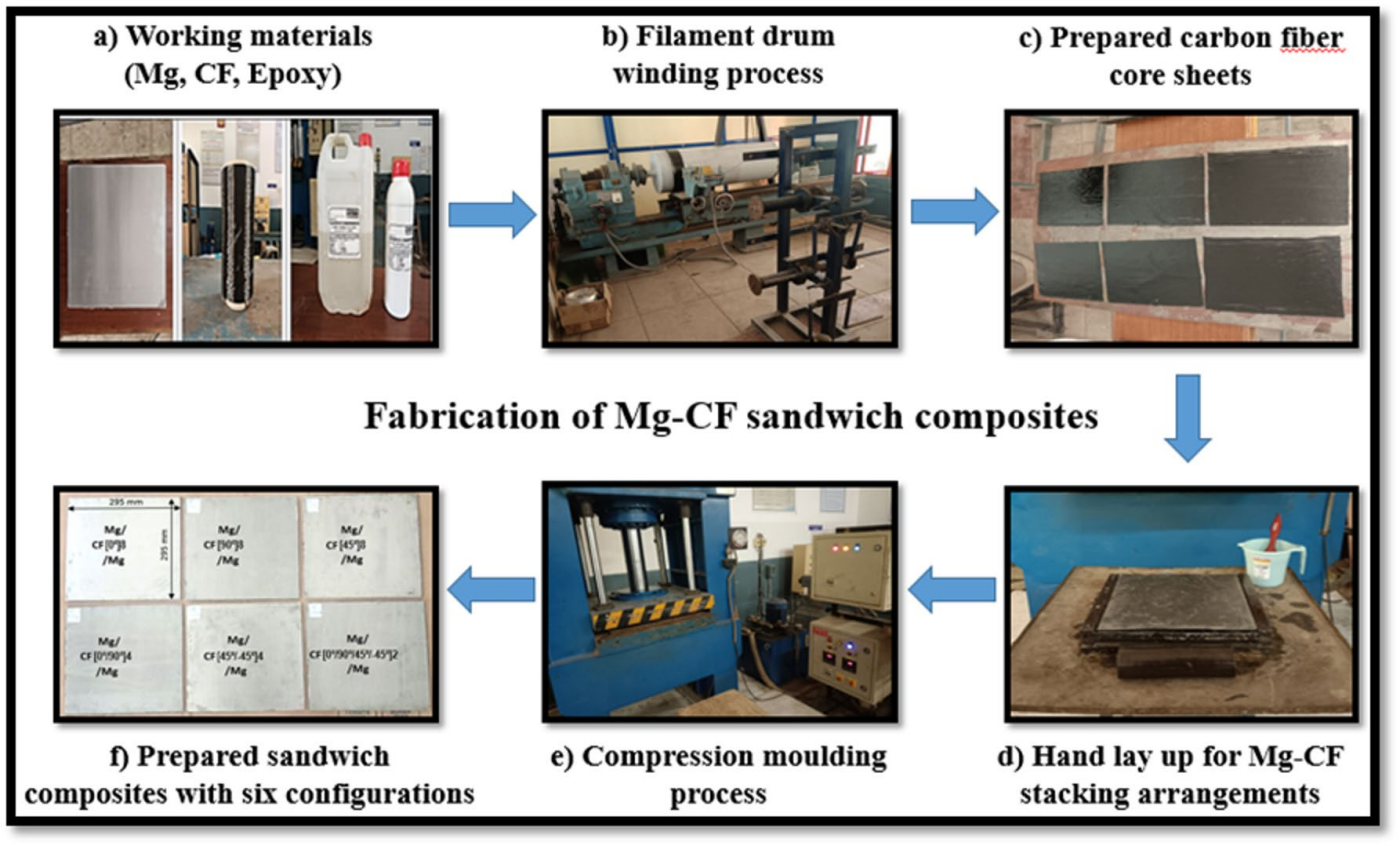
Systematic representation of the fabrication process for magnesium-carbon fiber-based sandwich composites.

The magnesium-carbon fiber (Mg-CF) sandwich composites were fabricated using a hybrid process combining filament drum winding, hand lay-up, and compression moulding to achieve controlled fiber orientation, uniform resin distribution, and strong interfacial bonding^41^. The sandwich structure consisted of two AZ31 magnesium alloy face sheets with nominal thicknesses of 0.5 mm each (top and bottom) and a carbon fiber-reinforced polymer (CFRP) core with a total thickness of approximately 2.2 mm, resulting in an overall laminate thickness of about 3.2 mm. Figure 1 illustrates the step-by-step fabrication sequence, including filament winding, resin impregnation, layer stacking, and final compression moulding used to produce the Mg-CF sandwich composites.

Initially, continuous carbon fiber tows were wound onto a cylindrical drum to obtain the prescribed fiber orientations corresponding to the desired stacking sequences. The wound fiber layers were subsequently removed from the drum and impregnated with a low-viscosity epoxy resin system using the hand lay-up technique^42^. Following winding, the laminates were allowed to cure at room temperature to form preliminary sheet structures, which were then cut into required orientations (0°, 90°, + 45°, and − 45°) and dimensions as per design requirements. These trimmed sheets were subsequently subjected to compression moulding at an elevated temperature of 80 °C for 2 h to enhance consolidation and resin flow. The CFRP core was constructed by stacking eight resin-impregnated carbon fiber sheets, each with an average thickness of approximately 0.22 mm, and then positioned between the two AZ31 magnesium face sheets to form a symmetric sandwich configuration. To improve fiber wetting and minimise void content, a hand roller was used to expel entrapped air and ensure uniform resin distribution throughout the laminate. The fiber volume fraction ($\:{V}_{f}$) of the composite was determined based on the rule of mixtures using the weight fractions and densities of the constituent materials. It is expressed as:

1 $$\:{V}_{f}=\frac{{W}_{f}/{\rho\:}_{f}}{\left({W}_{f}/{\rho\:}_{f}+{W}_{m}/{\rho\:}_{m}\right)}$$

where $\:{W}_{f}$and $\:{W}_{m}$represent the weight fractions of fiber and matrix, respectively, and $\:{\rho\:}_{f}$and $\:{\rho\:}_{m}$denote their corresponding densities. Based on the known material proportions used during fabrication, the fiber volume fraction was estimated to be approximately 55%.

The assembled lay-up was then transferred to a heated compression moulding press, where curing was carried out at a controlled temperature of 150 °C under a constant pressure of 35 bar for 2 h, followed by gradual cooling under sustained pressure to minimise residual stresses and ensure strong interfacial bonding at the magnesium face sheet and CFRP core interfaces^43^. The interfacial bonding between the magnesium face sheets and the epoxy matrix plays a critical role in ensuring structural integrity and efficient load transfer within the sandwich composite. In the present study, bonding is primarily achieved through mechanical interlocking and adhesive interaction facilitated by the epoxy resin. Surface preparation of the magnesium sheets, including cleaning and mild abrasion, enhances adhesion by increasing surface roughness and promoting improved resin wetting. The cured epoxy matrix enables effective stress transfer between the carbon fiber core and magnesium face sheets; however, due to the inherent dissimilarity between metallic and polymeric phases, the interface may be susceptible to localised debonding under mechanical or tribological loading.

**Fig. 2:**
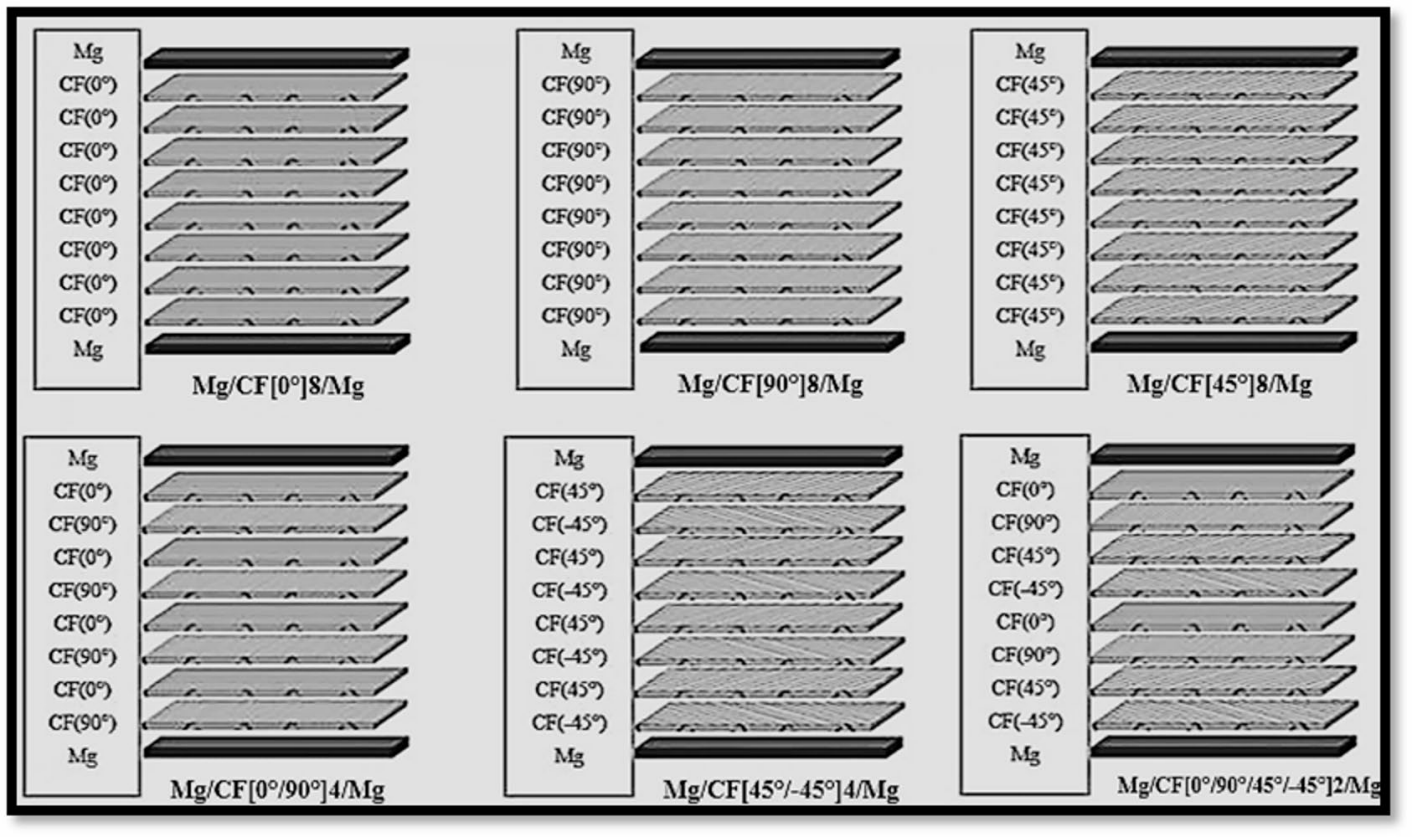
Schematic representation of sandwich laminates with six different configurations.

Based on classical laminate plate theory, six distinct stacking configurations, namely unidirectional ([0°], [45°], and [90°]), bidirectional ([0°/90°] and [45°/−45°]), and multidirectional ([0°/90°/45°/−45°]), were fabricated to analyse the influence of fiber orientation on surface and tribological performance^44^. Figure 2 illustrates the schematic representation of the Mg-CF sandwich laminates with six different stacking configurations used in this study. The stacking sequences and configuration details of the fabricated magnesium-carbon fiber sandwich composite laminates are summarised in Table 3.

**Table 3:** Stacking sequence and configuration details of prepared Mg-CF sandwich composite laminates.

S. No	Laminate configuration	Fiber orientations	Stacking sequences
1	Unidirectional	0°	Mg/CF[0°]_8_/Mg
2		90°	Mg/CF[90°]_8_/Mg
3		45°	Mg/CF[45°]_8_/Mg
4	Bidirectional	0°/90°	Mg/CF[0°/90°]_4_/Mg
5		45°/−45°	Mg/CF[45°/−45°]_4_/Mg
6	Multidirectional	0°/90°/45°/−45°	Mg/CF[0°/90°/45°/−45°]_2_/Mg

### Sample preparation

**Fig. 3:**
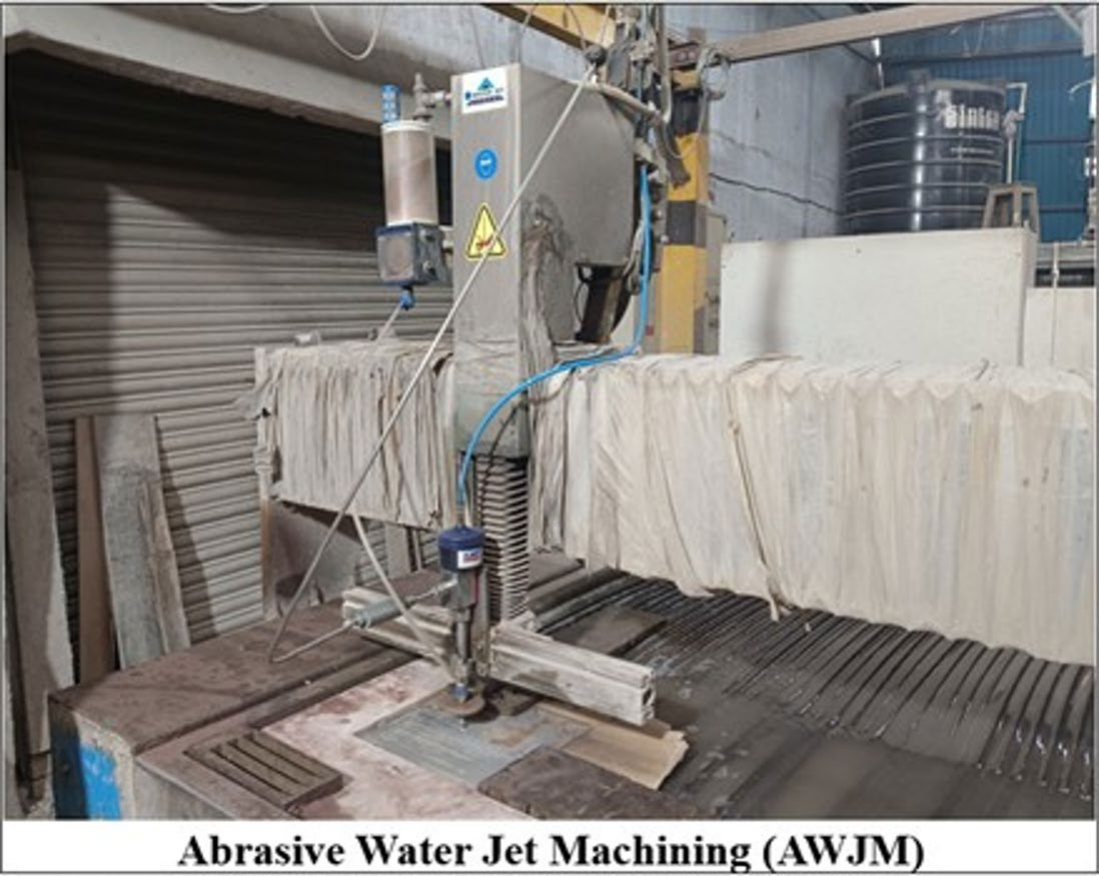
Preparation of samples using abrasive water jet machining (AWJM) process.

The fabricated composite panels were machined into test specimens in accordance with relevant ASTM standards to ensure dimensional accuracy and repeatability. All specimens for tribological and microhardness evaluations were prepared using abrasive water jet machining (AWJM), as shown in Fig. 3.

**Fig. 4:**
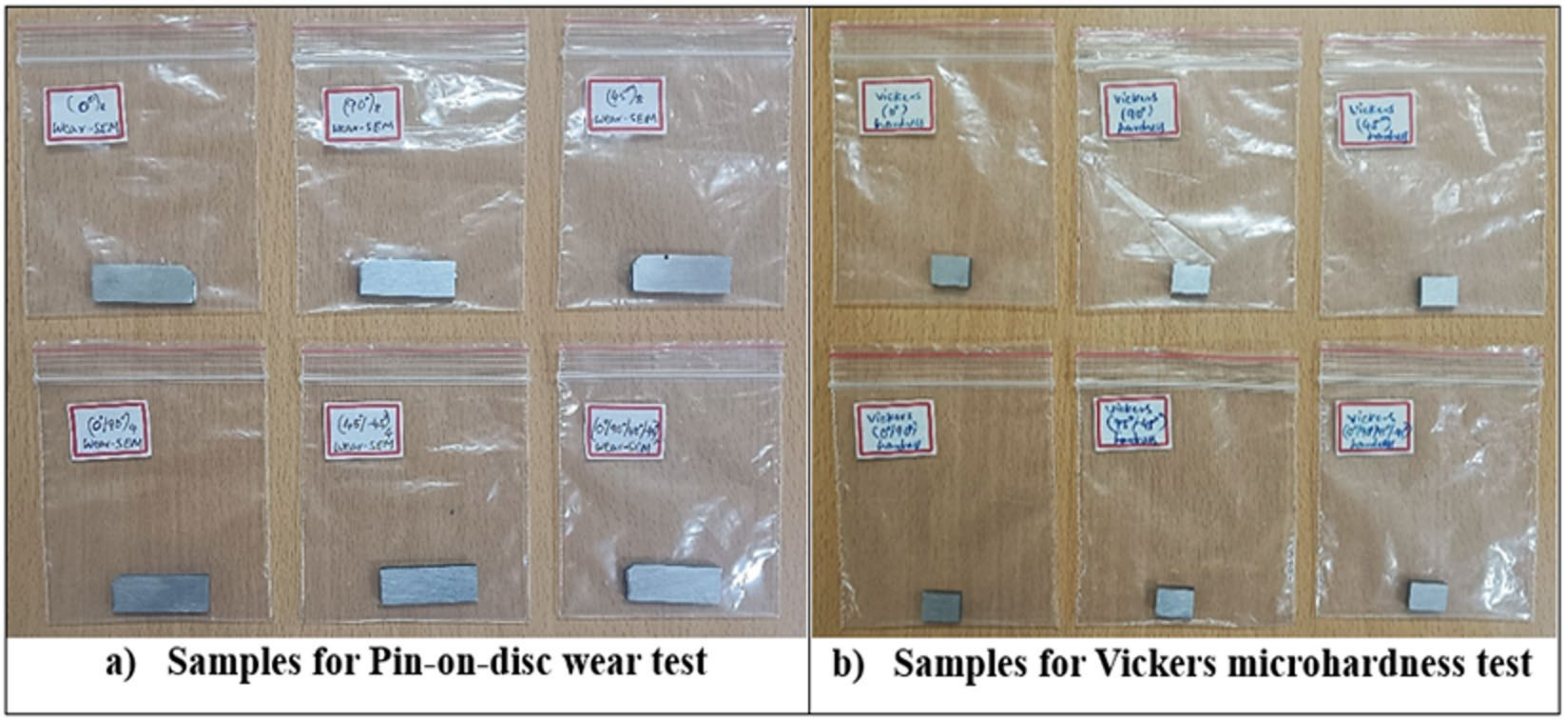
Prepared magnesium-carbon fiber sandwich composite specimens for (a) pin-on-disc wear testing and (b) Vickers microhardness testing.

Machining was carried out on an S3015 system with a cutting bed of 3 m × 1.5 m, using 80-mesh garnet abrasive at a flow rate of 400 g/min and a maximum pressure of 4200 bar. A diamond orifice and carbide nozzle ensured stable jet formation and dimensional precision. The traverse speed was maintained at 700 mm/min, and the stand-off distance was controlled within 0.5–3 mm to minimise kerf taper and improve edge quality^45^. For pin-on-disc wear testing (ASTM G99), specimens of 30 mm × 10 mm × 3.2 mm were prepared, with the cross-sectional surface selected as the sliding interface to evaluate interfacial wear behaviour^46^. The surfaces were sequentially ground using 1000, 1500, and 2000 grit silicon carbide papers to obtain a uniform finish while avoiding fiber pull-out and interfacial damage. Prior to testing, all specimens underwent identical grinding and polishing procedures to ensure consistent surface conditions. Although the initial roughness was not quantitatively measured, uniform preparation was maintained to minimise variability in tribological response. For Vickers microhardness testing (ASTM E384), the magnesium face sheets and cross-sectional surfaces were polished to a mirror finish^47^. All specimens were ultrasonically cleaned in ethanol and dried under ambient conditions prior to testing. The prepared specimens are shown in Fig. 4.

## Testing and characterization

### Pin-on-disc wear test

**Fig. 5:**
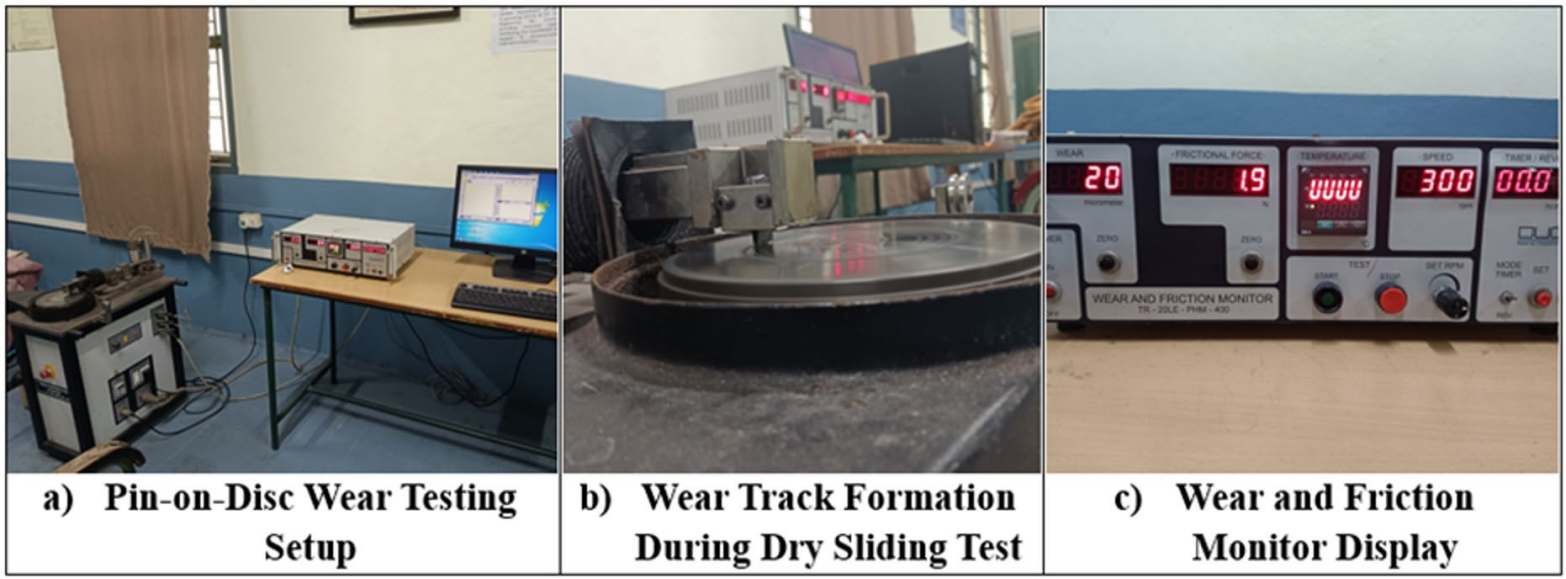
Pin-on-disc wear testing and friction monitoring system (DUCOM-TR-20LE-PHM-400).

The tribological behaviour of the magnesium-carbon fiber (Mg-CF) sandwich composites was evaluated using a pin-on-disc tribometer (Wear and Friction Monitor, DUCOM-TR-20LE-PHM-400) under dry sliding conditions in accordance with ASTM G99^48^. This configuration was selected to simulate service-relevant sliding contact and to enable a systematic investigation of the influence of fiber orientation on wear performance and frictional stability. The wear test was conducted on the cross-sectional surface of the Mg-CF composite laminates to directly assess the combined response of the magnesium face sheets, carbon fiber-reinforced polymer (CFRP) core, and their interfacial regions under sliding contact. Figure 5 illustrates the pin-on-disc wear testing setup, wear track formation during sliding, and the real-time friction monitoring system used for evaluating tribological performance.

The specimens were sectioned perpendicular to the laminate plane using a precision cutting machine to expose the cross-section. The surfaces were then sequentially ground using silicon carbide (SiC) abrasive papers of progressively finer grit sizes, followed by polishing with diamond paste to obtain a smooth and defect-free finish. Special care was taken during preparation to avoid fiber pull-out, matrix smearing, and interfacial damage, ensuring that the measured tribological response accurately reflects intrinsic material behaviour. Prior to testing, all specimens were cleaned using acetone to remove surface contaminants and ensure consistent contact conditions.

This cross-sectional testing approach enables the capture of edge-related wear mechanisms, interfacial debonding behaviour, and fiber-matrix interactions under sliding conditions, which are not accessible in conventional surface-based wear studies. The selected test parameters, such as normal load of 20 N, sliding velocity of approximately 1.41 m/s (300 rpm, 90 mm track diameter), and test duration of 10 min, were chosen based on commonly reported conditions in the literature for magnesium-based and fiber-reinforced composite systems under moderate dry sliding regimes^49^.

### Surface roughness testing

Surface roughness measurements were carried out using a contact profilometer (Taylor Hobson Surtronic S-128) in accordance with ISO 4287 and ISO 4288 standards to quantify surface degradation induced by sliding wear^50^. Prior to measurement, the instrument was calibrated using a certified reference standard to ensure accuracy and repeatability. The polished cross-sectional surface of each specimen, corresponding to the sliding contact interface, was selected as the measurement plane. This approach enables direct evaluation of surface damage at the magnesium-CFRP interface and within the composite core, where fibre exposure, matrix smearing, and interfacial degradation are most prominent.

**Fig. 6:**
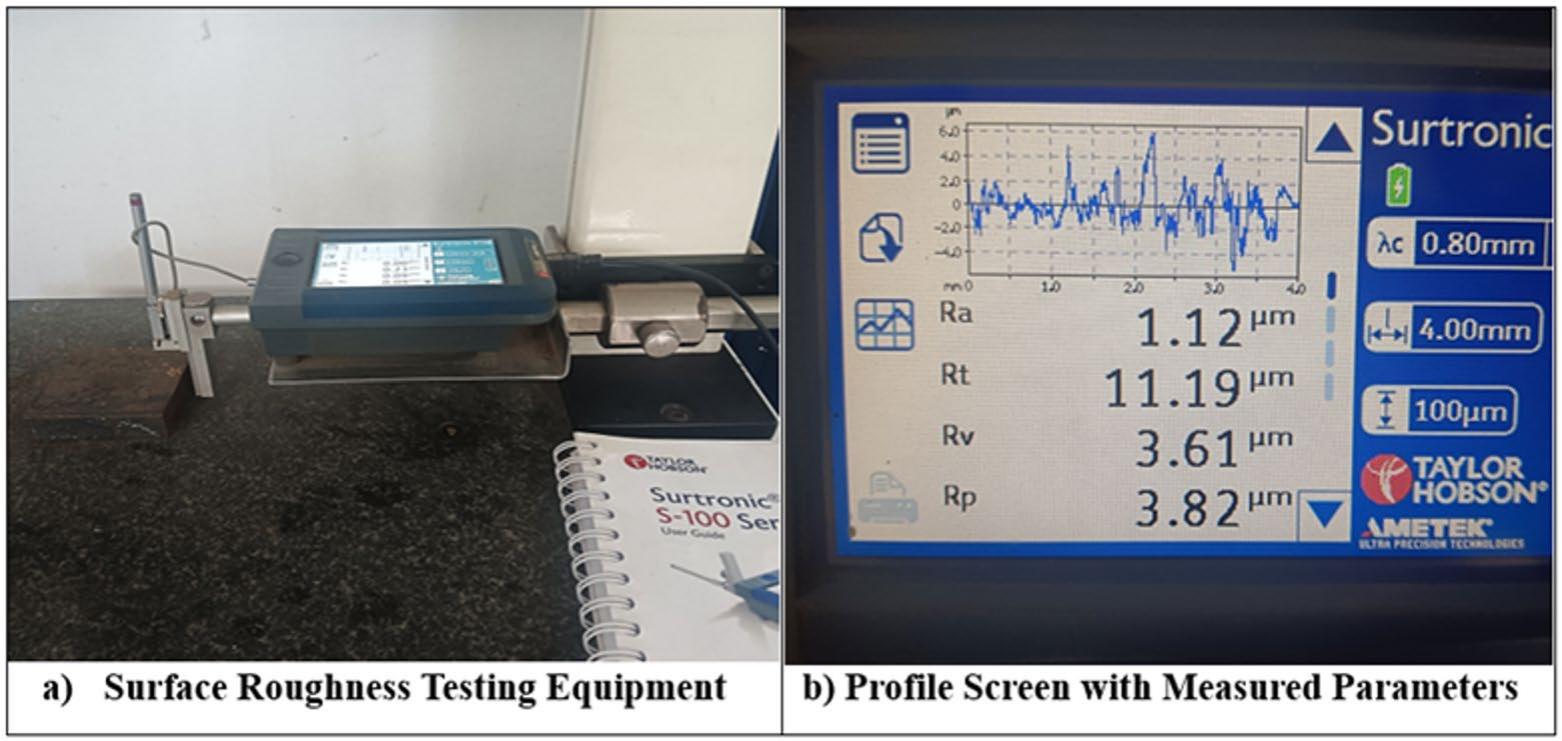
Surface roughness measurement using stylus profilometer (Taylor Hobson Surtronic S-128).

For each specimen, multiple measurements were taken along the wear track to ensure statistical reliability, and the average roughness parameters (Ra, Rq, and Rz) were recorded. The difference between pre-wear and post-wear roughness values (ΔRa) was used to quantify the extent of surface degradation. Figure 6 presents the surface roughness measurement setup along with a representative roughness profile showing key parameters before and after wear testing.

### Vickers microhardness testing

The Vickers microhardness of the Mg-CF sandwich composites was measured using a micro-Vickers hardness tester (H-1000) in accordance with ASTM E384^51^. Hardness measurements were performed on both the magnesium face sheet surfaces and the polished cross-sectional plane to evaluate surface resistance and the influence of fiber orientation and interfacial architecture across the laminate thickness. All specimens were prepared to a mirror-like finish to ensure well-defined indentation impressions and minimise measurement errors. A load of 100 g with a dwell time of 15 s was applied for all hardness measurements to ensure consistency and comparability across different configurations^52^.

**Fig. 7:**
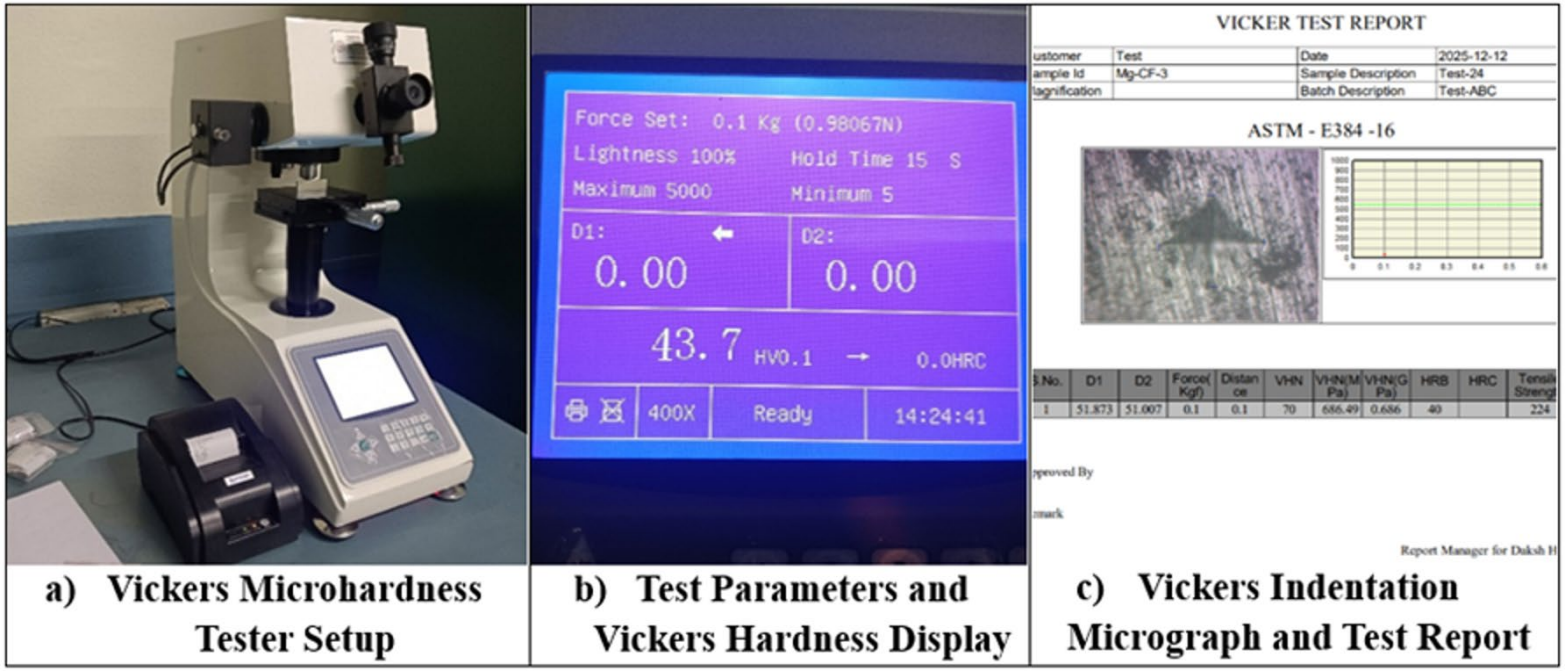
Vickers microhardness testing (H-1000) and indentation analysis.

For cross-sectional analysis, indentations were placed normal to the laminate thickness, covering the magnesium face sheets, CFRP core, and interfacial regions. This enabled the assessment of depth-dependent hardness variation and load-transfer characteristics within the sandwich structure. The indentation diagonals were measured using the integrated optical microscope, and hardness values were calculated accordingly. Multiple indentations were performed for each region, and average values were reported to ensure reliability. Figure 7 shows the micro-Vickers hardness testing setup, indentation measurement interface, and representative test output.

### Scanning electron microscopy (SEM)

The worn cross-sectional surfaces of the Mg-CF sandwich composites were analysed using a TESCAN VEGA3 SBH scanning electron microscope (SEM) to identify dominant wear mechanisms and microstructural features^53^. Prior to imaging, specimens were ultrasonically cleaned to remove loose debris, mounted on conductive stubs, and sputter-coated with a thin conductive layer to minimise charging effects. SEM observations were carried out at multiple magnifications to capture detailed morphological features, including ploughing grooves, matrix smearing, fiber exposure, fiber pull-out, crack formation, and interfacial debonding.

**Fig. 8:**
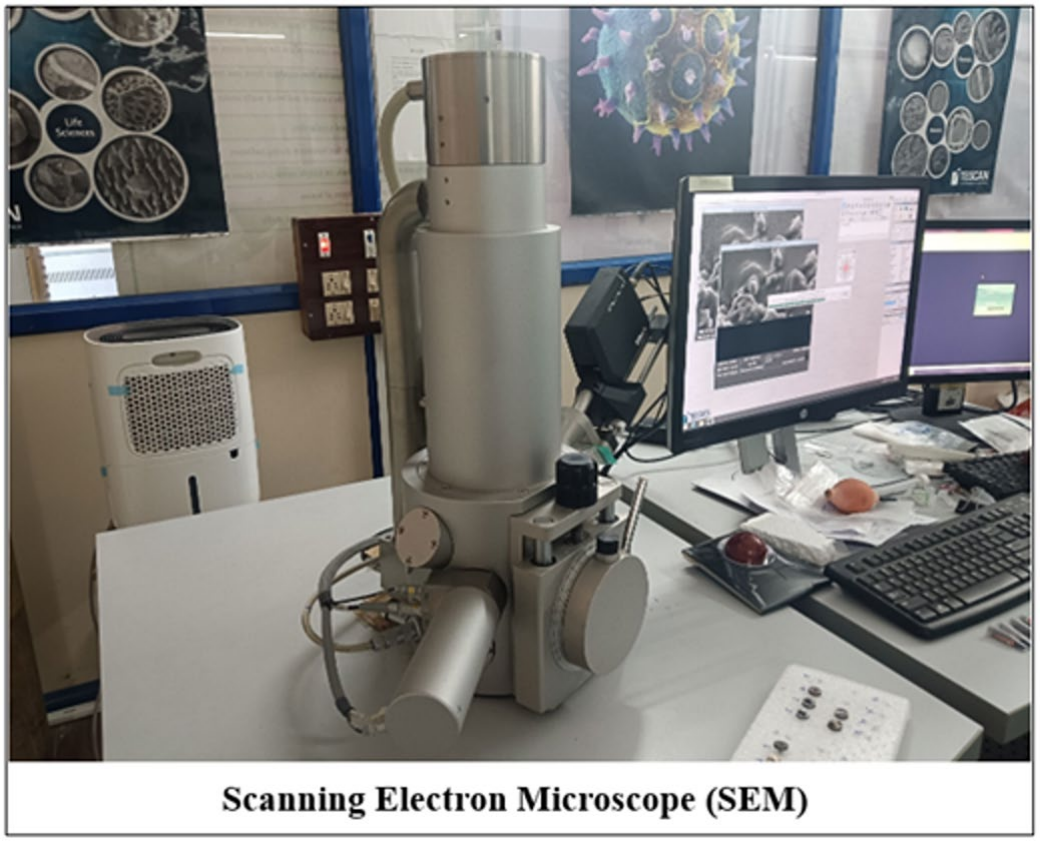
Scanning electron microscope (SEM) used for microstructural examination.

Special attention was given to the magnesium-CFRP interface and core regions to understand the influence of fiber orientation and stacking sequence on wear damage evolution. The SEM observations were systematically correlated with quantitative tribological results, including wear rate, surface roughness, and hardness measurements, to establish structure-property-tribology relationships. Figure 8 presents the SEM instrument used for microstructural analysis and representative imaging conditions.

## Results and discussions

The results are systematically discussed by correlating fibre architecture (structure) with mechanical and tribological responses (properties) to establish clear structure-property relationships in the Mg-CF sandwich composites.

### Tribological performance (wear rate and friction behaviour)

The tribological behaviour of the Mg-CF sandwich composites was evaluated under a constant normal load of 20 N and a rotational speed of 300 rpm, with a wear track diameter of 90 mm, corresponding to a linear sliding velocity of approximately 1.41 m/s. Each configuration was tested for a duration of 10 min, resulting in a total sliding distance of approximately 848 m. The applied normal load (20 N) and rotational speed (300 rpm) were selected based on ASTM G99 guidelines and established literature on Mg-CFRP tribological systems, representing a moderate sliding regime suitable for stable wear evaluation without excessive thermal effects^54,55^. For each laminate configuration, three independent experiments were conducted, and the reported values represent the mean ± standard deviation. The sliding contact area was defined by the exposed cross-sectional surface of the specimen (12.5 mm × 3.2 mm), ensuring consistent contact geometry across all configurations. This cross-sectional testing approach enables direct evaluation of the combined tribological response of the magnesium face sheets, CFRP core, and their interfacial regions under sliding conditions, providing a more realistic representation of multi-material interaction compared to conventional surface wear testing. The specific wear rate and coefficient of friction were analysed to evaluate the influence of fiber orientation and stacking sequence on the tribological performance of Mg-CF sandwich composites.

#### Specific wear rate

The wear rate of the Mg-CF sandwich composite configurations was determined using the mass loss method, wherein the specimens were weighed before and after each wear test to quantify material removal under dry sliding conditions. The density of the composite specimens used in wear rate calculations was estimated using the rule of mixtures based on the densities and volume fractions of the constituent materials. The measured mass loss values for all configurations, along with their average values, are summarised in Table 4, while the calculated volume loss and corresponding specific wear rate are presented in Table 5.

**Table 4:** Mass loss of magnesium-carbon fiber sandwich composites with different fiber stacking configurations measured over three wear runs under constant normal load.

Samples configurations	Initial weight (grams)	Mass loss (g) for Run 1	Mass loss (g) for Run 2	Mass loss (g) for Run 3	Avg. Mass loss (g)	Final weight (g)
Mg/CF[0°]_8_/Mg	2.341	0.027	0.033	0.027	0.029	2.254
Mg/CF[90°]_8_/Mg	2.255	0.042	0.041	0.046	0.043	2.126
Mg/CF[45°]_8_/Mg	2.094	0.035	0.037	0.038	0.037	1.984
Mg/CF[0°/90°]_4_/Mg	2.336	0.036	0.030	0.034	0.033	2.236
Mg/CF[45°/−45°]_4_/Mg	2.079	0.024	0.026	0.028	0.026	2.001
Mg/CF[0°/90°/45°/−45°]_2_/Mg	2.285	0.021	0.025	0.026	0.024	2.213

The total sliding distance (S) was calculated as:

2 $$\:S=\:\pi\:DNt$$

where.

D = wear track diameter (mm) = 90.

N = rotational speed (rpm) = 300.

t = test duration (min) = 10.

The volume loss (V) was obtained from gravimetric measurements:

3 $$\:V=\frac{{\Delta\:}m}{\rho\:}$$

where.

Δm = Avg. mass loss (g).

ρ = Effective composite density (g/mm³) = 1.65 g/cm^3^.

The specific wear rate (k) was determined using:

4 $$\:K=\frac{V}{{F}_{n}\times\:S}$$

where.

* V* = volume loss (mm³).

$\:{F}_{n}$ = Applied normal load (N) = 20.

* S*= sliding distance (m).

The uncertainty in specific wear rate was estimated by propagating the standard deviation of mass loss through the volume loss and wear rate calculations.

**Table 5:** Avg. mass loss, volume loss, and specific wear rate of magnesium-carbon fiber sandwich composites under dry sliding conditions.

Samples configurations	Avg. mass loss (g)	Avg. volume loss (mm³)	Avg. specific wear rate (×10⁻³ mm³/*N*·m)
Mg/CF[0°]_8_/Mg	0.029 ± 0.00346	17.57 ± 2.10	(1.04 ± 0.124) ×10⁻³
Mg/CF[90°]_8_/Mg	0.043 ± 0.00265	26.06 ± 1.60	(1.54 ± 0.094) ×10⁻³
Mg/CF[45°]_8_/Mg	0.037 ± 0.00153	22.22 ± 0.93	(1.31 ± 0.055) ×10⁻³
Mg/CF[0°/90°]_4_/Mg	0.033 ± 0.00306	20.20 ± 1.85	(1.19 ± 0.109) ×10⁻³
Mg/CF[45°/−45°]_4_/Mg	0.026 ± 0.00200	15.76 ± 1.21	(0.93 ± 0.071) ×10⁻³
Mg/CF[0°/90°/45°/−45°]_2_/Mg	0.024 ± 0.00265	14.55 ± 1.60	(0.86 ± 0.094) ×10⁻³

**Fig. 9:**
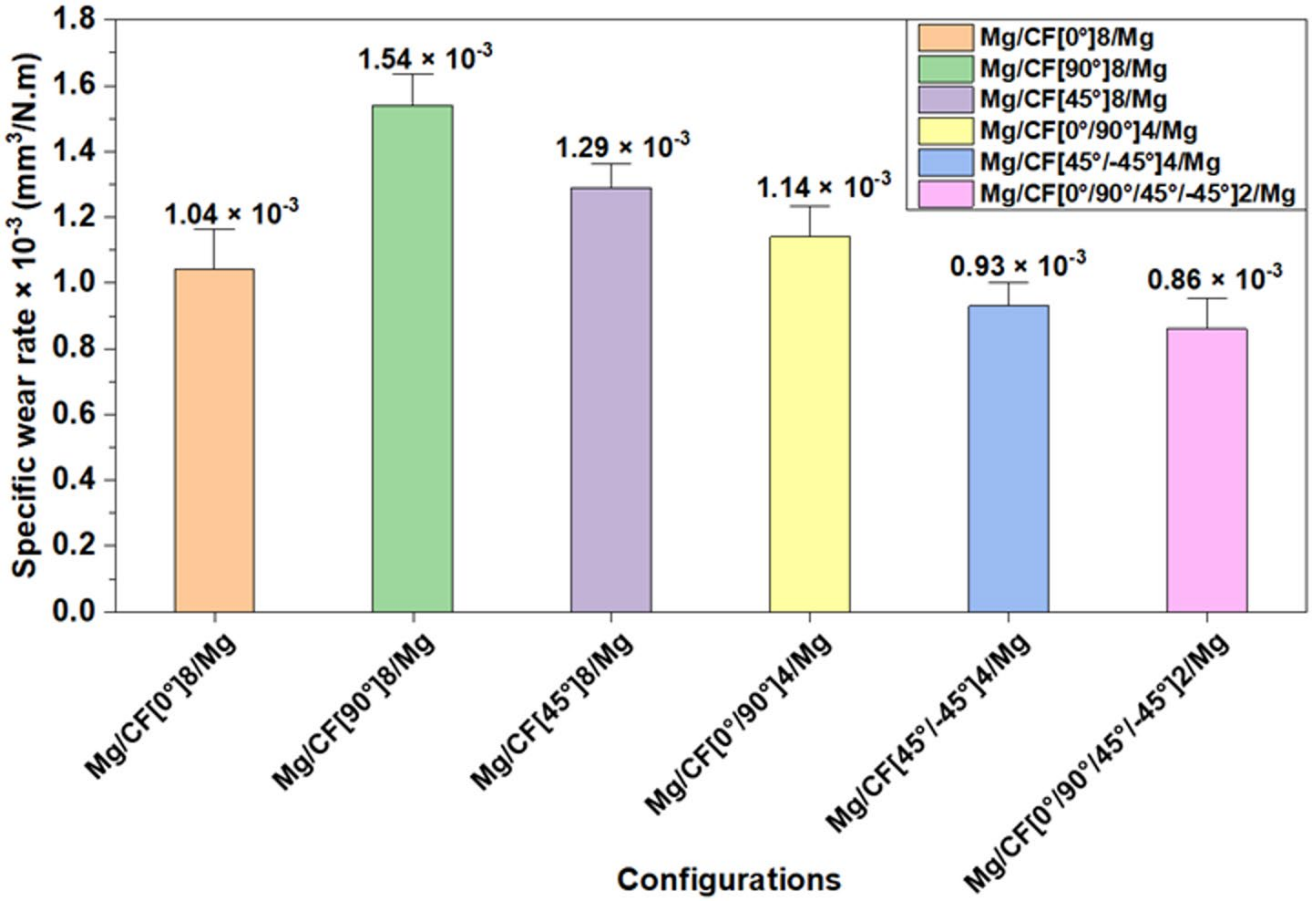
Bar graph representation of average specific wear rate for six Mg-CF sandwich composite configurations.

Figure 9 presents a comparative representation of the average specific wear rate for all configurations. Error bars representing ± standard deviation (*n* = 3) are included in the figures to indicate the variability and repeatability of the experimental measurements.

The results reveal a strong dependence of wear behaviour on fiber orientation and stacking architecture. Among all configurations, the Mg/CF[90°]_8_/Mg laminate exhibited the highest mass loss (0.043 g), volume loss (26.06 mm³), and specific wear rate (1.54 mm³/N·m), indicating inferior wear resistance. This behaviour is primarily attributed to fiber alignment perpendicular to the sliding direction, which limits effective load transfer and promotes matrix-dominated wear, leading to increased material removal. The inclusion of standard deviation in wear rate values ensures statistical reliability of the reported tribological trends. Among all tested orientations, the multidirectional laminate (Mg/CF[0°/90°/45°/−45°]_2_/Mg) exhibited the lowest specific wear rate of 0.86 × 10⁻³ mm³/N·m, representing an improvement of approximately 44% compared to the unidirectional configuration. This result clearly demonstrates that fiber architecture plays a decisive role in governing tribological performance.

The wear trend, arranged in order of increasing specific wear rate, is as follows:

$$\:Mg/CF\left[0^\circ\:/90^\circ\:/45^\circ\:/-45^\circ\:]/Mg<Mg/CF[45^\circ\:/-45^\circ\:]/Mg<Mg/CF[0^\circ\:]/Mg<Mg/CF[0^\circ\:/90^\circ\:]/Mg<Mg/CF[45^\circ\:]/Mg<Mg/CF[90^\circ\:\right]/Mg$$

The observed differences in wear performance can be explained based on the underlying load-transfer mechanisms and fiber-matrix interactions. Multidirectional laminates exhibit superior tribological behaviour due to their ability to distribute applied stresses more uniformly across multiple fiber orientations, thereby reducing localised stress concentrations at the sliding interface. This behaviour is supported by SEM observations (Sect. 5.3), which show smoother wear tracks, reduced fibre pull-out, and limited matrix cracking in multidirectional laminates. These features indicate improved interfacial stability and suggest mechanisms such as fibre bridging that contribute to enhanced wear resistance. In contrast, unidirectional laminates, particularly with fibers oriented perpendicular to the sliding direction, exhibit anisotropic load transfer, leading to increased matrix-dominated wear, fiber debonding, and higher material removal.

Furthermore, fiber alignment relative to the sliding direction plays a critical role in governing wear mechanisms such as debris formation, interfacial stress distribution, and transfer film stability. Fibers oriented perpendicular to the sliding direction are more susceptible to microfracture and debonding, resulting in unstable debris generation and increased wear. In contrast, multidirectional configurations promote effective load sharing and facilitate stable transfer film formation, as qualitatively inferred from SEM observations of smoother wear tracks and reduced debris agglomeration (Sect. 5.3). Additionally, strong fiber-matrix interfacial bonding enhances load transfer and suppresses subsurface crack propagation, whereas weak bonding leads to fiber pull-out and matrix removal^56,57^. However, these mechanisms are interpreted qualitatively based on surface morphology, and detailed quantitative characterization was not performed in the present study.

**Fig. 10:**
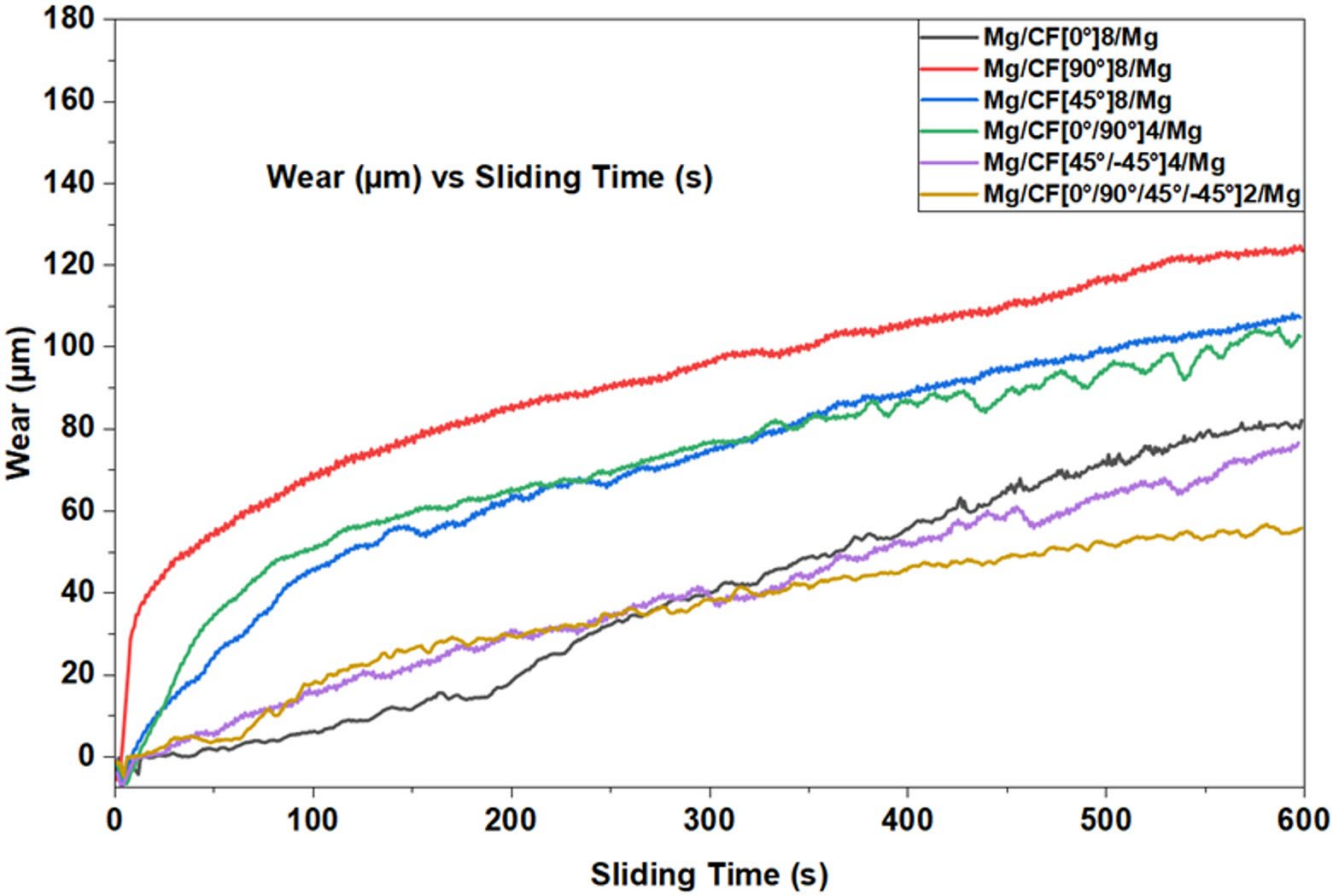
Wear (µm) vs. sliding time (s) curves for magnesium-carbon fiber sandwich composites with six different fiber orientations under a normal load.

The average wear versus sliding time curves obtained from the WINDUCOM software (Fig. 10) illustrate the progressive material removal behaviour of all laminate configurations under constant loading conditions. Multidirectional laminates demonstrate a more stable wear progression, while unidirectional configurations exhibit greater fluctuations, signifying diminished tribological stability.

**Fig. 11:**
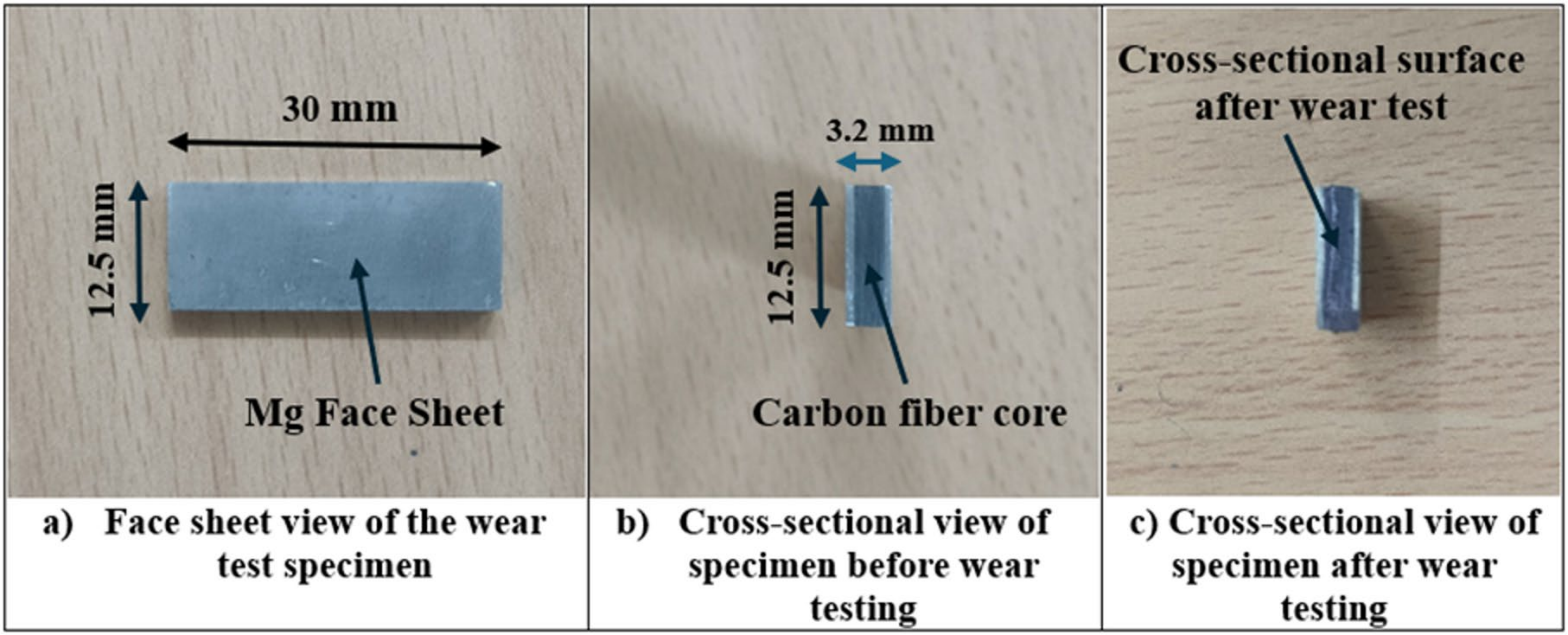
Wear test specimen geometry and cross-sectional condition before and after sliding.

These wear trends are further supported by the corresponding variations in surface roughness and hardness, where configurations exhibiting lower wear also demonstrate reduced roughness evolution and higher resistance to localised deformation. This confirms that the tribological behaviour of Mg-CF sandwich composites is governed by the combined influence of fiber architecture, interfacial integrity, and mechanical response under sliding conditions. Figure 11 presents the wear test specimen geometry and cross-sectional conditions before and after sliding.

#### Frictional force and coefficient of friction

The frictional behaviour of the magnesium-carbon fiber (Mg-CF) sandwich composites was evaluated by analysing the steady-state region of the frictional force-time curves obtained during the pin-on-disc tests. To ensure reliability and consistency, only the stabilised portion of the curve, corresponding to the post-running-in stage, was considered for calculating the representative frictional force and coefficient of friction (COF). The average frictional force and corresponding coefficient of friction were determined using the following relations:

Calculate average frictional force (FF)

5 $$\:{F}_{f}=\frac{1}{n}\sum\:_{i=1}^{n}{F}_{i}$$

where.

$\:{F}_{i}$= instantaneous friction force values (N) obtained from the steady-state region of the friction force–time curve.

* n*= number of data points.

The instantaneous coefficient of friction (COF) was calculated as:

6 $$\:\mu\:=\:\frac{{F}_{f}}{{F}_{n}}$$

where.

$\:{F}_{f}$ = average frictional force (N).

$\:{F}_{n}$ = applied normal load (N) = 20.

Standard Deviation

7 $$\:SD=\sqrt{\frac{\sum\:_{i=1}^{n}({F}_{i}-\stackrel{\prime }{F}{)}^{2}}{n-1}}$$

where.

$\:\stackrel{\prime }{F}$ = mean frictional force (N).

95% Confidence Interval (CI)

8 $$\:CI=\stackrel{\prime }{x}\pm\:\frac{t\cdot\:SD}{\sqrt{n}}$$

where.

*n* = 3.

t ≈ 4.303 (for 95% CI).

**Table 6:** Average frictional force, standard deviation, and coefficient of friction of Mg-CF sandwich composites under a 20 N normal load.

Samples configurations	Avg. friction force (*N*)	Coefficient of friction (µ)	95% CI (µ)
Mg/CF[0°]_8_/Mg	4.05 ± 0.18	0.203 ± 0.009	0.203 ± 0.022
Mg/CF[90°]_8_/Mg	7.25 ± 0.3	0.363 ± 0.015	0.363 ± 0.037
Mg/CF[45°]_8_/Mg	5.4 ± 0.28	0.27 ± 0.014	0.27 ± 0.035
Mg/CF[0°/90°]_4_/Mg	5.05 ± 0.35	0.253 ± 0.0175	0.253 ± 0.043
Mg/CF[45°/−45°]_4_/Mg	2.95 ± 0.4	0.148 ± 0.02	0.148 ± 0.050
Mg/CF[0°/90°/45°/−45°]_2_/Mg	2.9 ± 0.23	0.145 ± 0.0115	0.145 ± 0.029

To assess statistical reliability, 95% confidence intervals were calculated based on three independent measurements. The limited overlap between confidence intervals indicates statistically meaningful differences in friction behaviour among the laminate configurations. The average frictional force and corresponding COF for the investigated laminates are presented in Table 6. The results clearly indicate that frictional response is strongly dependent on fiber orientation and interfacial load-sharing characteristics. Among all configurations, the Mg/CF[90°]_8_/Mg laminate exhibited the highest frictional force (7.25 ± 0.3 N) and COF (0.363), indicating unstable sliding conditions and increased interfacial resistance. This behaviour is primarily attributed to fiber alignment perpendicular to the sliding direction, which promotes fiber debonding, matrix-dominated deformation, and enhanced ploughing action during sliding. In contrast, the multidirectional Mg/CF[0°/90°/45°/−45°]_2_/Mg laminate recorded the lowest frictional force (2.9 ± 0.23 N) and COF (0.145), reflecting smoother sliding behaviour and improved tribological compatibility. Similarly, the Mg/CF[45°/−45°]_4_/Mg configuration also exhibited relatively low friction (2.95 ± 0.4 N, µ = 0.148), confirming that balanced fiber orientations contribute to reduced frictional instability and improved load distribution.

**Fig. 12:**
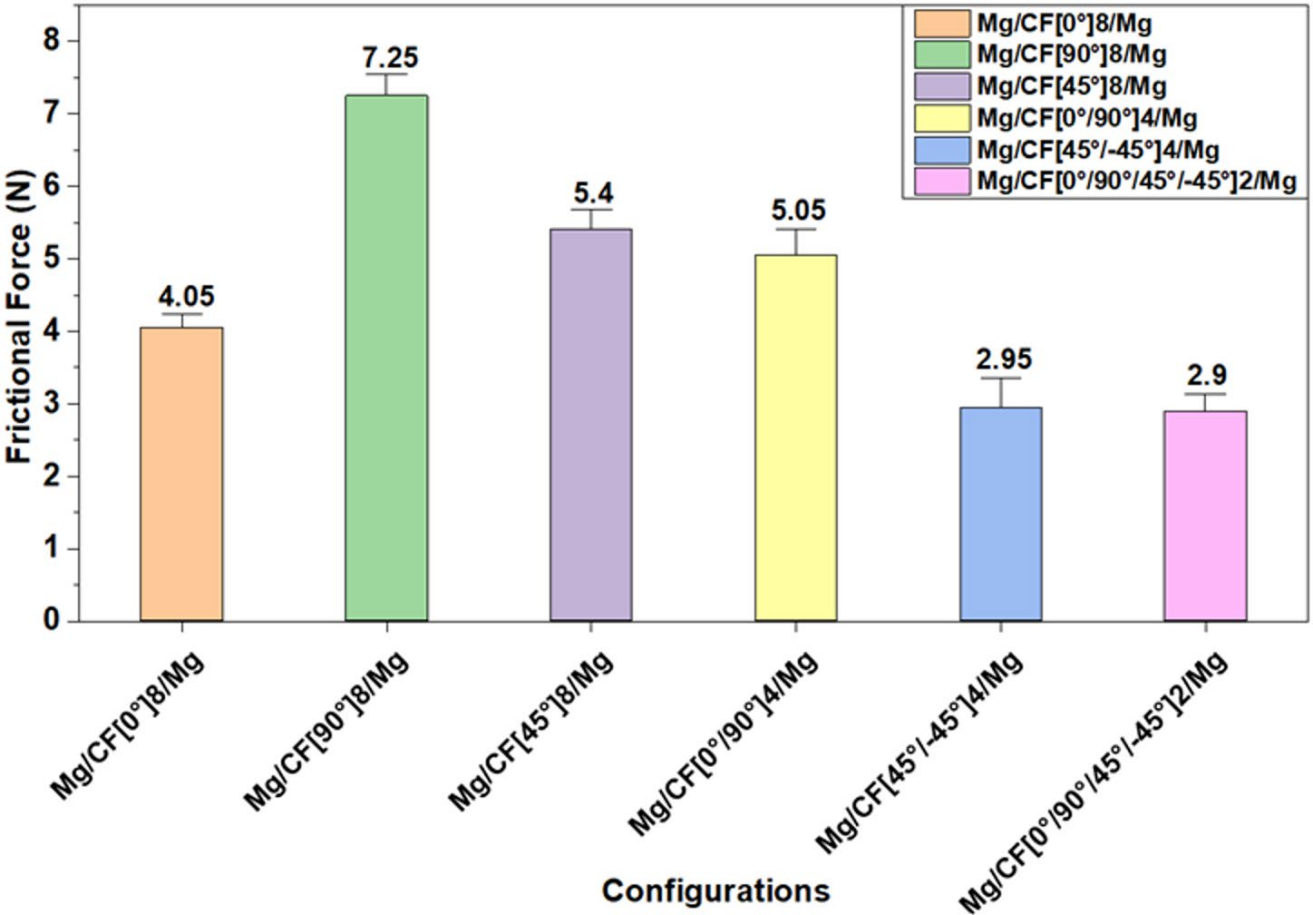
Bar graph representation of average frictional force for six Mg-CF sandwich composite configurations under dry sliding conditions.

**Fig. 13:**
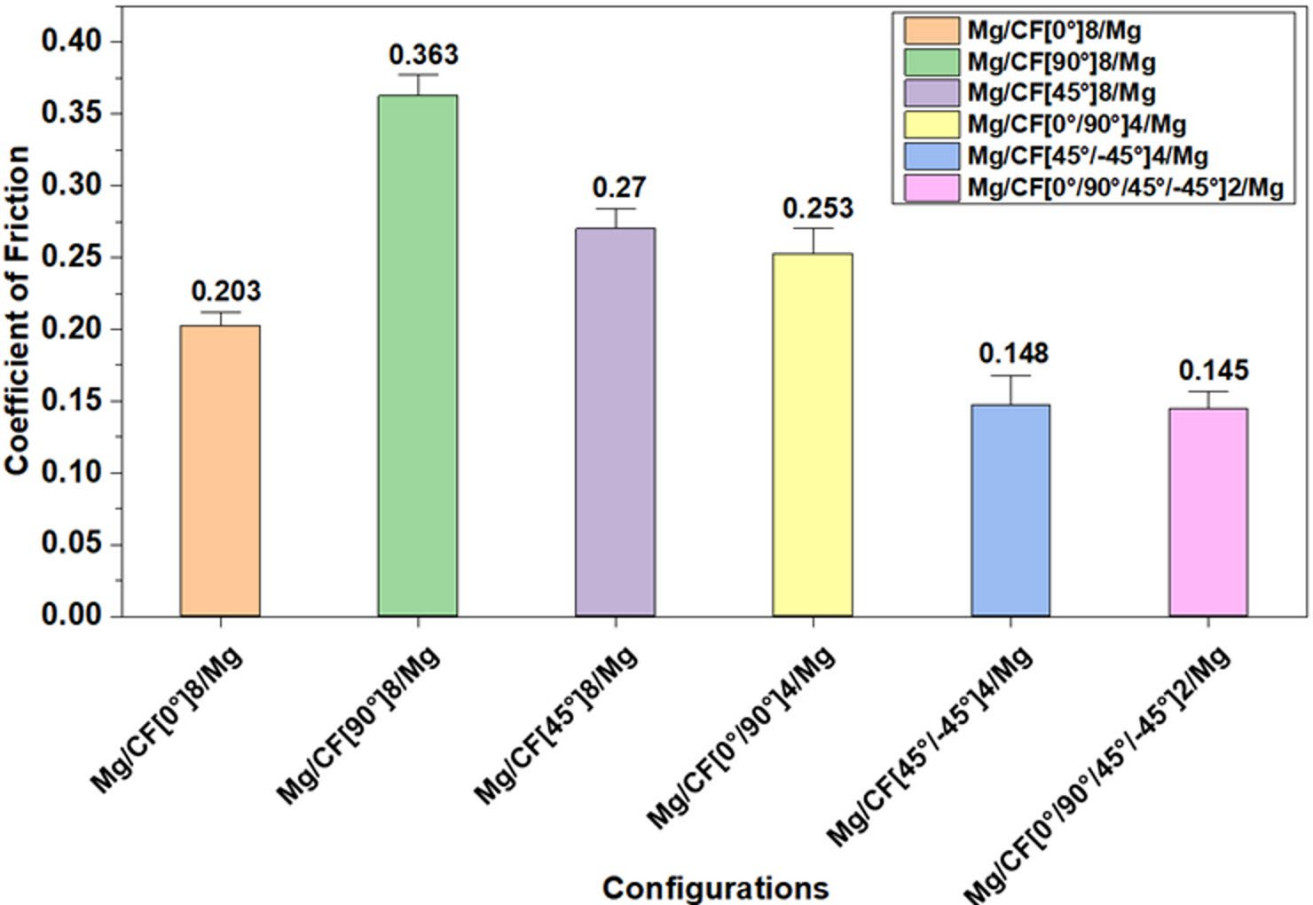
Bar graph representation of the average coefficient of friction (COF) for Mg-CF sandwich composite configurations.

Figure 12 presents a comparative bar graph of the average frictional force for all Mg-CF sandwich configurations, highlighting the influence of stacking architecture on frictional performance, while Fig. 13 illustrates the corresponding average coefficient of friction, showing the variation in frictional behaviour with respect to fiber orientation and stacking sequence. The inclusion of error bars in the frictional force and coefficient of friction plots reflects the consistency of repeated measurements and demonstrates good experimental repeatability.

To further validate the observed trends, a one-way ANOVA was conducted at a 95% confidence level for both the specific wear rate and coefficient of friction. The analysis yielded statistically significant results, with p-values of 1.28 × 10⁻⁵ for wear rate and 3.71 × 10⁻⁹ for COF. For the coefficient of friction, an F-value of F = 92.52 (df = 5, 12) was obtained, which is significantly higher than the critical value (Fcrit = 3.11), confirming that the effect of fiber architecture on tribological behaviour is statistically significant.

**Fig. 14:**
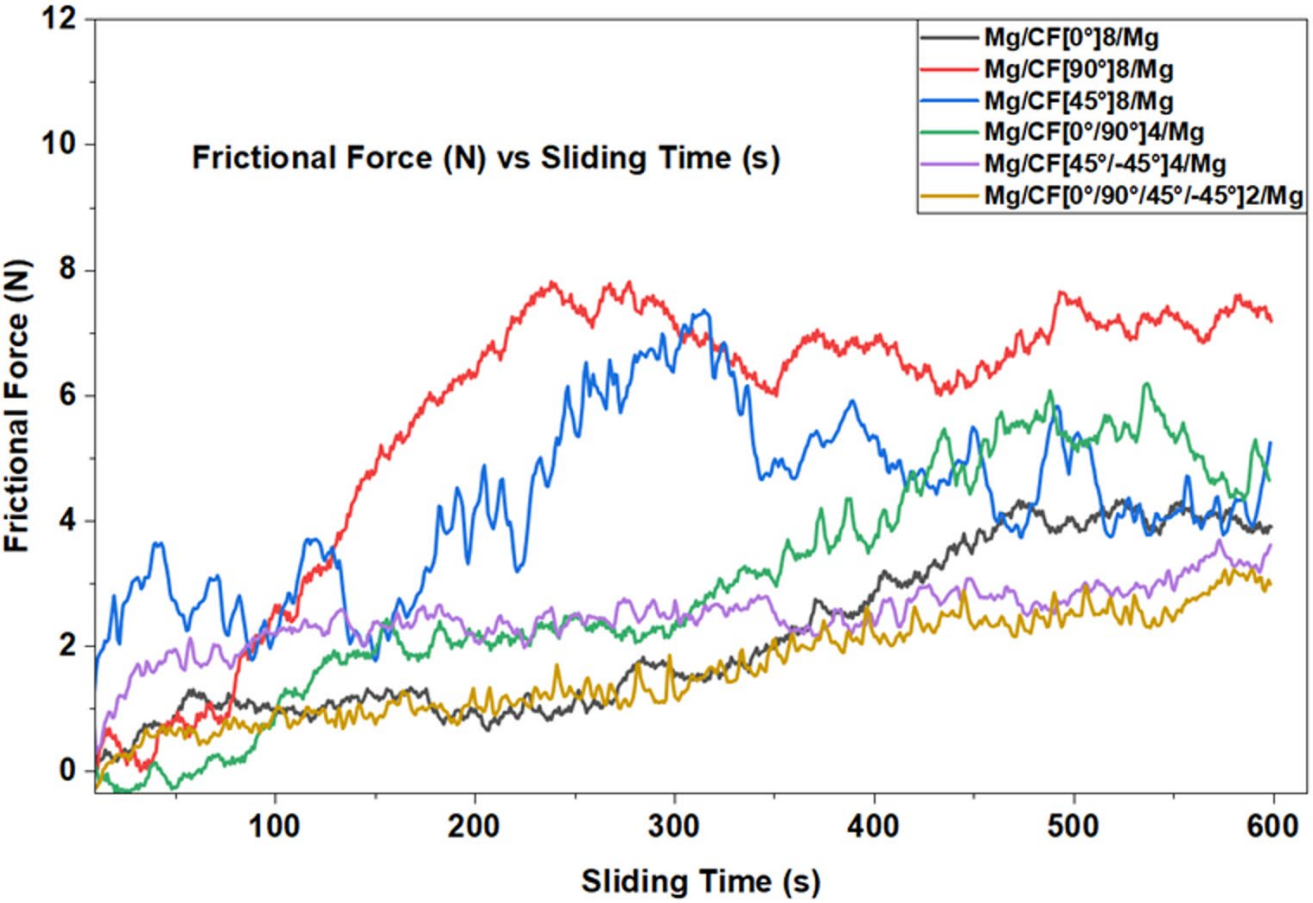
Frictional force (N) vs. Sliding time (s) curves for Six Mg-CF sandwich composite configurations under dry sliding conditions.

Figure 14 presents the average frictional force versus sliding time curves for the six Mg-CF sandwich composite configurations, providing insight into both steady-state and transient (running-in) behaviour. During the initial stage of sliding, all configurations exhibit a rapid rise in frictional force associated with asperity interaction, surface adaptation, and initial material removal. This running-in regime is followed by a relatively stabilised region where frictional fluctuations become more consistent. Multidirectional laminates, particularly Mg/CF[0°/90°/45°/−45°]_2_/Mg, demonstrate smoother and more stable friction profiles with reduced fluctuations, indicating the formation of a stable transfer layer and improved interfacial compatibility. In contrast, the Mg/CF[90°]_8_/Mg configuration exhibits higher fluctuations and instability during the transient stage, attributed to fiber debonding, irregular debris generation, and intermittent third-body interactions at the sliding interface.

It is important to note that the relationship between hardness and wear resistance is not strictly linear, particularly in polymer-based composite systems. The frictional and wear behaviour is also governed by dynamic factors such as debris formation, third-body interactions, and thermal effects during sliding. Wear debris may act either as an abrasive medium, increasing material removal, or as a compacted transfer layer that reduces direct surface contact and stabilises friction. Additionally, friction-induced localised heating at the sliding interface may contribute to matrix softening and influence the dominant wear mechanisms. However, temperature evolution during sliding was not directly measured in the present study, and therefore these effects are interpreted qualitatively based on the observed wear morphology and tribological response. Therefore, the tribological performance of Mg-CF sandwich composites is governed by a synergistic interaction between fiber orientation, interfacial bonding, hardness, and evolving surface conditions during sliding.

The overall tribological behaviour of the Mg-CF sandwich composites is governed by the combined influence of fiber architecture, hardness, and surface roughness evolution. Configurations with higher core hardness, particularly multidirectional laminates, exhibit enhanced resistance to localised deformation and material removal, resulting in lower wear rates and improved frictional stability. Conversely, laminates with lower hardness show increased susceptibility to plastic deformation, fiber pull-out, and interfacial damage, leading to higher wear and unstable frictional response. Surface roughness evolution further reflects the severity of wear mechanisms, where smoother worn surfaces correspond to more stable sliding behaviour. Overall, these results confirm that fiber orientation plays a decisive role in governing frictional performance, with multidirectional configurations demonstrating superior stability and reduced interfacial resistance compared to unidirectional laminates.

### Cross-sectional surface roughness measurements

Cross-sectional surface roughness measurements were conducted to quantify the extent of topographical modification induced by sliding wear. The post-wear roughness values were evaluated with reference to a uniformly prepared initial surface condition, enabling quantitative assessment of surface degradation. Following wear testing, specimens were sectioned to approximately 10 mm in length to facilitate stable mounting during profilometry while preserving the original contact face dimensions (12.5 mm × 3.2 mm). For each stacking configuration, surface traces were recorded at three spatially separated locations along the wear track to account for localised heterogeneity in surface damage. The stylus was traversed perpendicular to the sliding direction to capture representative asperity deformation and ploughing features generated during sliding. A cut-off length of 0.8 mm and an evaluation length of 4.0 mm were maintained for all measurements to ensure consistency.

The primary roughness parameter reported was the arithmetic mean roughness (Ra), while the root mean square roughness (Rq) and maximum peak-to-valley height (Rz) were also determined to provide additional insight into asperity distribution and severe surface damage characteristics. All roughness values are reported as mean ± standard deviation based on repeated measurements. The obtained data were correlated with wear rate and frictional behaviour to establish quantitative relationships between fiber architecture and surface integrity.

Average Roughness Values.

Pre-Wear

8 $$\:R{a}_{pre}=\frac{R{a}_{1}+R{a}_{2}+R{a}_{3}}{3}$$

Post-Wear

9 $$\:R{a}_{post}=\frac{R{a}_{1}+R{a}_{2}+R{a}_{3}}{3}$$

Roughness Change

10 $$\:{\Delta\:}Ra=R{a}_{post}-R{a}_{pre}$$

Post-Wear

11 $$\:R{q}_{post}=\frac{R{q}_{1}+R{q}_{2}+R{q}_{3}}{3}$$

Post-Wear

12 $$\:R{z}_{post}=\frac{R{z}_{1}+R{z}_{2}+R{z}_{3}}{3}$$

The evolution of surface roughness during sliding is closely associated with the dominant wear mechanisms at the contact interface. Configurations exhibiting higher wear rates show increased roughness due to abrasive action, fibre pull-out, and matrix degradation, leading to deeper grooves and irregular surface features. Fibre orientations unfavourable to the sliding direction promote interfacial debonding and material removal, whereas configurations with improved load distribution and interfacial stability facilitate smoother sliding and reduced roughness growth. Table 7.

**Table 7:** Pre- and post-wear arithmetic surface roughness (Ra) and roughness variation (ΔRa) of Mg-CF sandwich composite configurations under dry sliding conditions.

Configuration	Ra Pre (µm)	Ra Post (µm)	ΔRa (µm)
Mg/CF[0°]_8_/Mg	0.68 ± 0.06	1.43 ± 0.07	0.75 ± 0.09
Mg/CF[90°]_8_/Mg	0.85 ± 0.07	1.58 ± 0.10	0.73 ± 0.12
Mg/CF[45°]_8_/Mg	0.90 ± 0.05	1.81 ± 0.06	0.91 ± 0.08
Mg/CF[0°/90°]_4_/Mg	0.72 ± 0.09	1.24 ± 0.11	0.52 ± 0.14
Mg/CF[45°/−45°]_4_/Mg	0.75 ± 0.10	1.96 ± 0.13	1.21 ± 0.16
Mg/CF[0°/90°/45°/−45°]_2_/Mg	0.70 ± 0.03	1.12 ± 0.04	0.42 ± 0.05

**Fig. 15:**
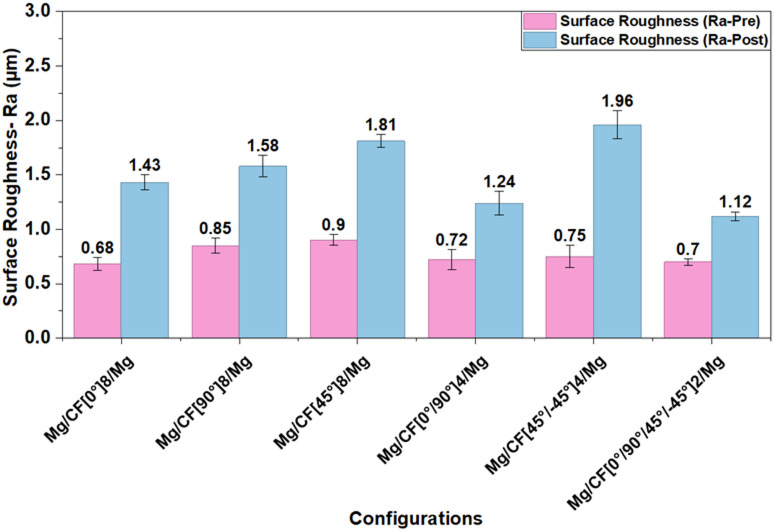
Comparison of surface roughness (Ra) values before and after wear testing for different Mg-CF sandwich laminate configurations.

**Fig. 16:**
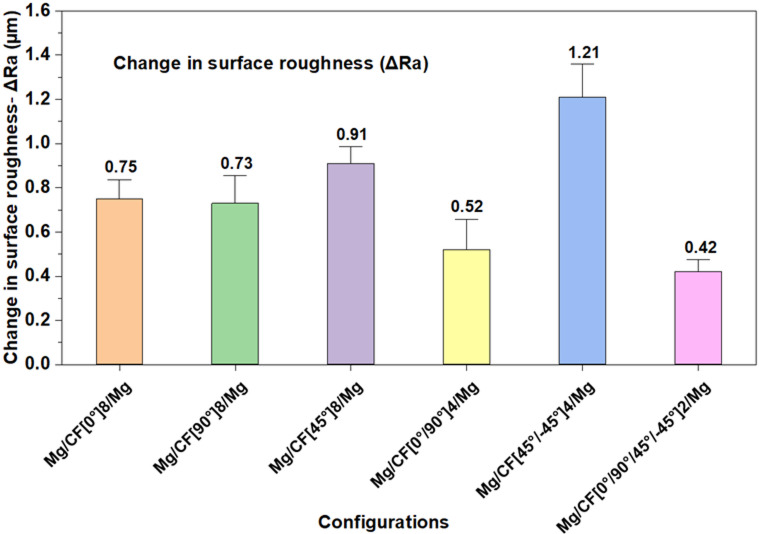
Variation in change in average surface roughness (ΔRa) for different Mg-CF sandwich laminate configurations after wear testing.

Figure 15 illustrates the variation in surface roughness (Ra) before and after wear testing, while Fig. 16 presents the corresponding roughness increment (ΔRa) for all configurations.

The results reveal a clear increase in cross-sectional roughness after wear for all laminate configurations, confirming progressive surface degradation under dry sliding conditions. The multidirectional laminate, Mg/CF[0°/90°/45°/−45°]_2_/Mg, exhibited the lowest post-wear roughness (Ra_post = 1.12 ± 0.04) and the smallest roughness increment (ΔRa = 0.42 ± 0.05 μm), indicating superior wear resistance and a stable sliding interface. The post-wear roughness parameters (Rq and Rz), summarised in Table 8, further confirm the severity of surface damage and the associated topographical changes induced by sliding wear.

**Table 8:** Post-wear surface roughness parameters (Rq and Rz) of Mg-CF sandwich composite configurations under dry sliding conditions.

Configuration	ΔRa (µm)	Rq Post (µm)	Rz Post (µm)
Mg/CF[0°]_8_/Mg	0.75 ± 0.09	2.23 ± 0.17	8.48 ± 0.87
Mg/CF[90°]_8_/Mg	0.73 ± 0.12	2.08 ± 0.21	9.04 ± 0.49
Mg/CF[45°]_8_/Mg	0.91 ± 0.08	3.65 ± 0.13	13.52 ± 1.21
Mg/CF[0°/90°]_4_/Mg	0.52 ± 0.14	1.88 ± 0.18	7.78 ± 0.82
Mg/CF[45°/−45°]_4_/Mg	1.21 ± 0.16	4.23 ± 0.10	16.89 ± 1.56
Mg/CF[0°/90°/45°/−45°]_2_/Mg	0.42 ± 0.05	1.47 ± 0.22	6.41 ± 1.14

**Fig. 17:**
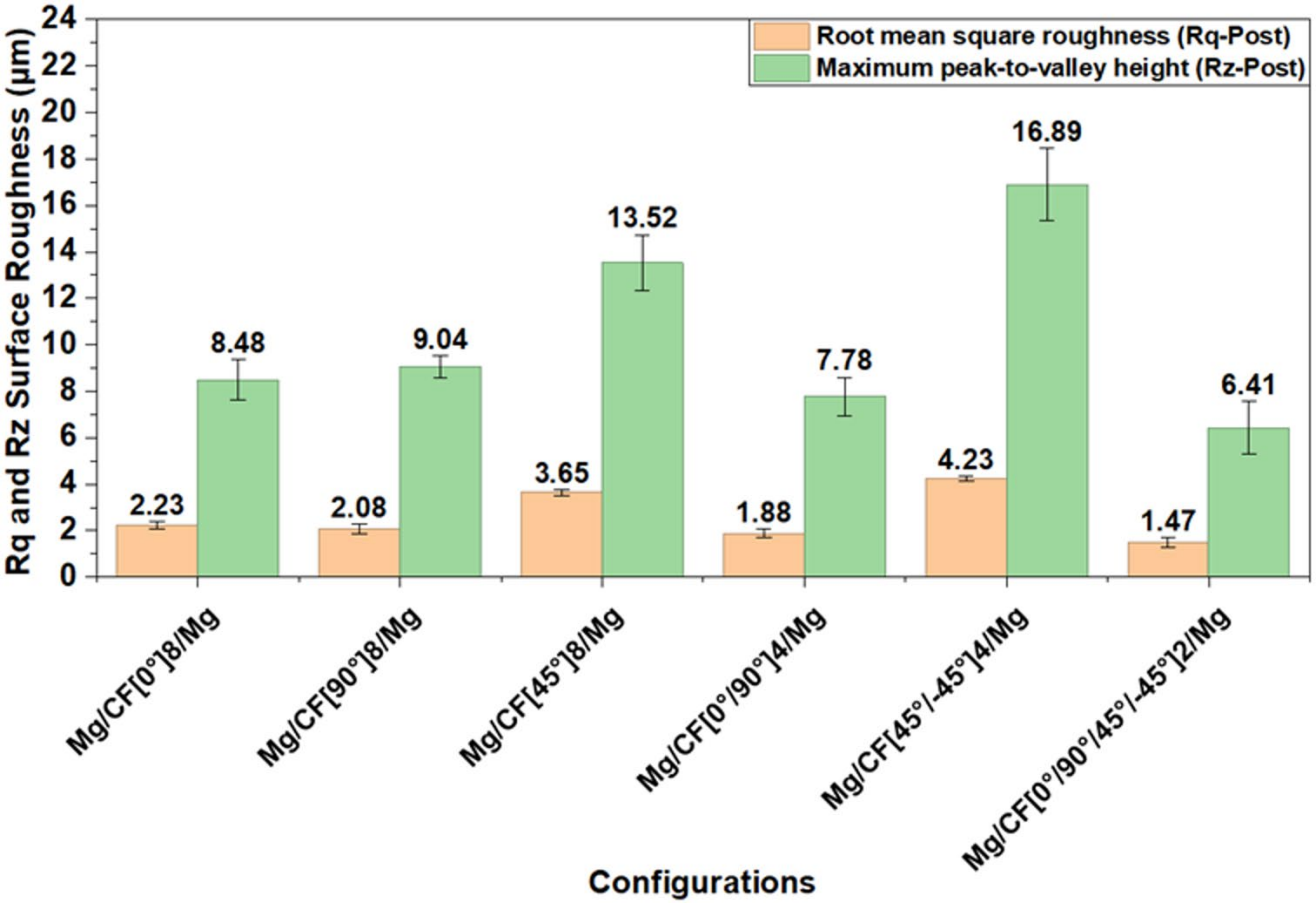
Comparison of post-wear surface roughness parameters (Rq and Rz) for different Mg-CF sandwich laminate configurations.

From Table 8, the multidirectional laminate configuration exhibits lower Rq (1.47 ± 0.22 μm) and Rz (6.41 ± 1.14 μm) values, suggesting limited groove formation and reduced peak-to-valley irregularities, which can be attributed to balanced load distribution and enhanced interfacial bonding. In contrast, the Mg/CF[45°/−45°]_4_/Mg configuration exhibited the most severe surface degradation, with Ra_post = (1.96 ± 0.13 μm) and a maximum roughness increment of ΔRa = 1.21 ± 0.16 μm. The elevated Rq (4.23 ± 0.10 μm) and Rz (16.89 ± 1.56 μm) values indicate pronounced ploughing, micro-cutting, and localised material removal. Similarly, the Mg/CF[45°]_8_/Mg laminate showed a considerable increase in roughness (ΔRa = 0.91 ± 0.08 μm), reflecting increased susceptibility to sliding-induced surface damage. Figure 17 compares these Rq and Rz values, highlighting the extent of peak-to-valley surface damage across different laminate architectures. Laminates with balanced fiber orientations exhibit lower roughness increments and more uniform wear tracks, whereas angle-ply configurations show higher Rz values, indicating deeper grooves and more severe interfacial damage. The results identify the multidirectional laminate as the dominant configuration exhibiting superior wear resistance and improved surface integrity, establishing fiber architecture as the key governing parameter in Mg-CF sandwich tribology.

### Surface morphology and SEM analysis

Scanning electron microscopy (SEM) was employed to investigate the wear-induced surface and subsurface damage mechanisms in magnesium-carbon fiber (Mg-CF) sandwich composites following pin-on-disc testing. The analysis encompasses both the cross-sectional morphology of the sandwich structure and the worn surface characteristics of the core along the sliding track. This combined approach enables a comprehensive assessment of deformation behaviour, interfacial integrity, and material removal mechanisms under dry sliding conditions. The SEM observations were systematically correlated with wear rate, coefficient of friction, surface roughness, and hardness results to establish meaningful structure-property relationships.

A semi-quantitative evaluation of key wear features was performed using scale calibration from the SEM micrographs. Parameters such as groove width, debris size, crack length, fibre pull-out length, and subsurface deformation depth were estimated across different laminate configurations under consistent magnification conditions. These measurements provide comparative insight into the severity of wear and the influence of fibre architecture on deformation behaviour. In addition, representative wear features, including micro-ploughing grooves, debris agglomeration, fibre exposure, interfacial damage, and surface cracking, were clearly identified and annotated in the SEM images to enhance interpretability and reproducibility. The combined qualitative and semi-quantitative assessment enables a consistent correlation between observed wear mechanisms and experimentally measured tribological performance.

#### Cross-sectional layered morphology after wear

**Fig. 18:**
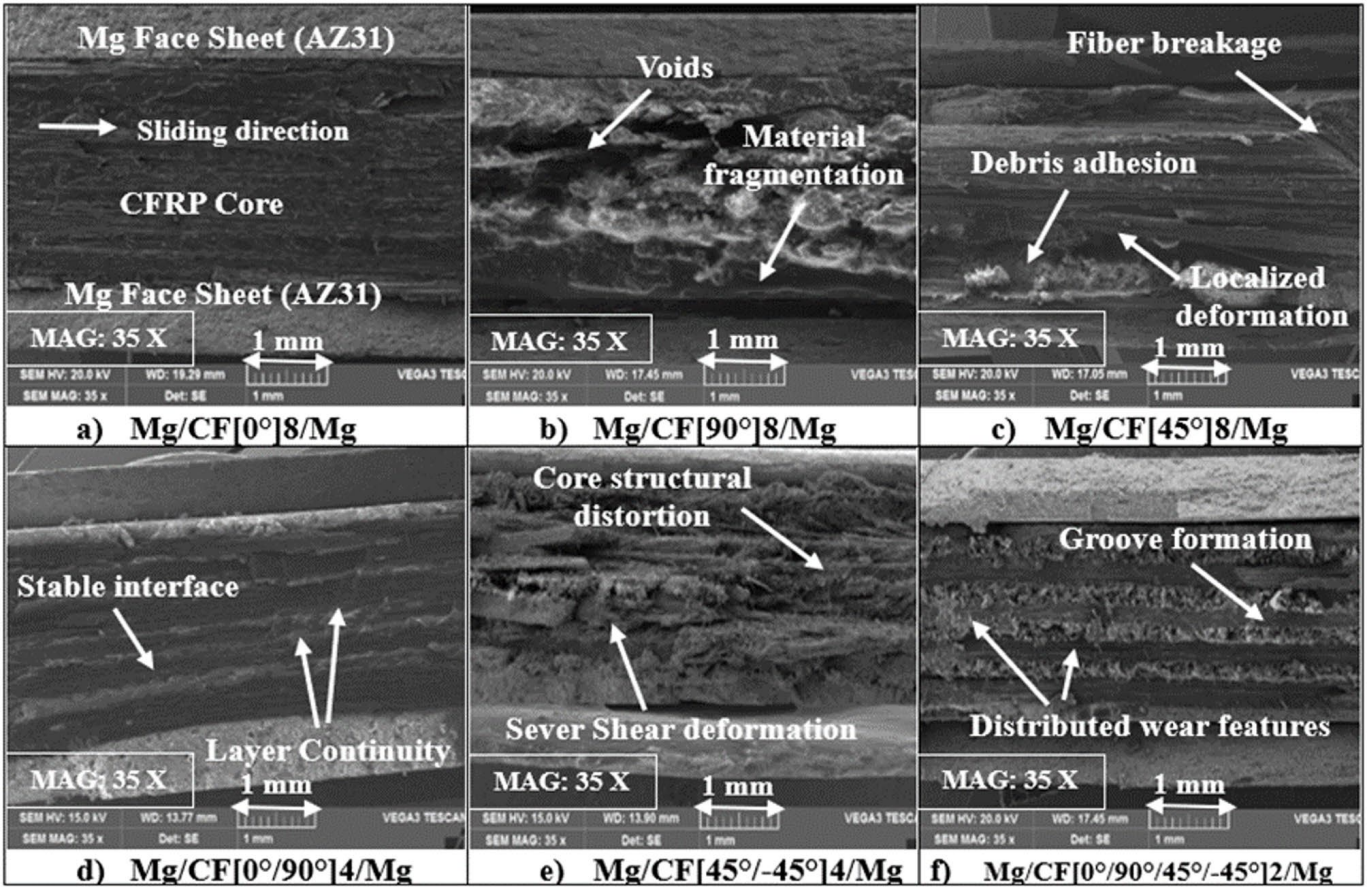
Cross-sectional SEM micrographs of worn Mg-CF sandwich composites showing the architecture for different stacking configurations.

Figure 18 presents the cross-sectional SEM micrographs of the worn Mg-CF sandwich composites, clearly revealing the three-layer architecture consisting of the upper magnesium face sheet, the CFRP core, and the lower magnesium face sheet after sliding wear. This three-layer architecture is retained across all stacking configurations, confirming that the overall structural integrity of the laminates is preserved under the applied sliding conditions. However, the extent of subsurface deformation, interfacial damage, and internal structural distortion varies significantly depending on fibre orientation and stacking sequence.

In the Mg/CF[0°]_8_/Mg configuration, the magnesium face sheet exhibits mild plastic deformation with a subsurface deformation depth of approximately 25–40 μm, accompanied by shallow penetration into the CFRP core (~ 15–25 μm), indicating relatively stable load transfer and limited interfacial disruption. In contrast, the Mg/CF[90°]_8_/Mg laminate shows pronounced interfacial degradation, characterised by void formation and material fragmentation within the core region, with feature sizes ranging from approximately 60 to 120 μm and an overall damage zone extending up to ~ 150 μm. This significant subsurface damage reflects severe stress concentration and inefficient load transfer associated with transverse fibre orientation. The Mg/CF[45°]_8_/Mg configuration exhibits intermediate behaviour, with localised deformation zones of approximately 60–90 μm and noticeable debris adhesion at the interface, suggesting moderate wear severity. Improved interfacial stability is observed in the Mg/CF[0°/90°]_4_/Mg laminate, where deformation depths are comparatively lower (~ 30–50 μm), indicating enhanced stress redistribution due to the hybrid fibre arrangement. In contrast, the Mg/CF[45°/−45°]_4_/Mg configuration displays severe internal distortion within the CFRP core, with shear deformation zones extending up to approximately 180–260 μm, reflecting poor resistance to shear-induced damage and interfacial instability.

The multidirectional Mg/CF[0°/90°/45°/−45°]_2_/Mg laminate demonstrates the most uniform cross-sectional response, with controlled subsurface deformation in the range of ~ 30–60 μm and evidence of debris compaction without large-scale delamination. Overall, the cross-sectional analysis indicates that the severity of deformation and interfacial damage decreases significantly with increasing fibre orientation complexity. Multidirectional laminates exhibit improved stress distribution and interfacial stability, whereas unidirectional configurations, particularly the 90° orientation, are more susceptible to subsurface damage and structural degradation.

#### Worn surface morphology of CFRP core

**Fig. 19:**
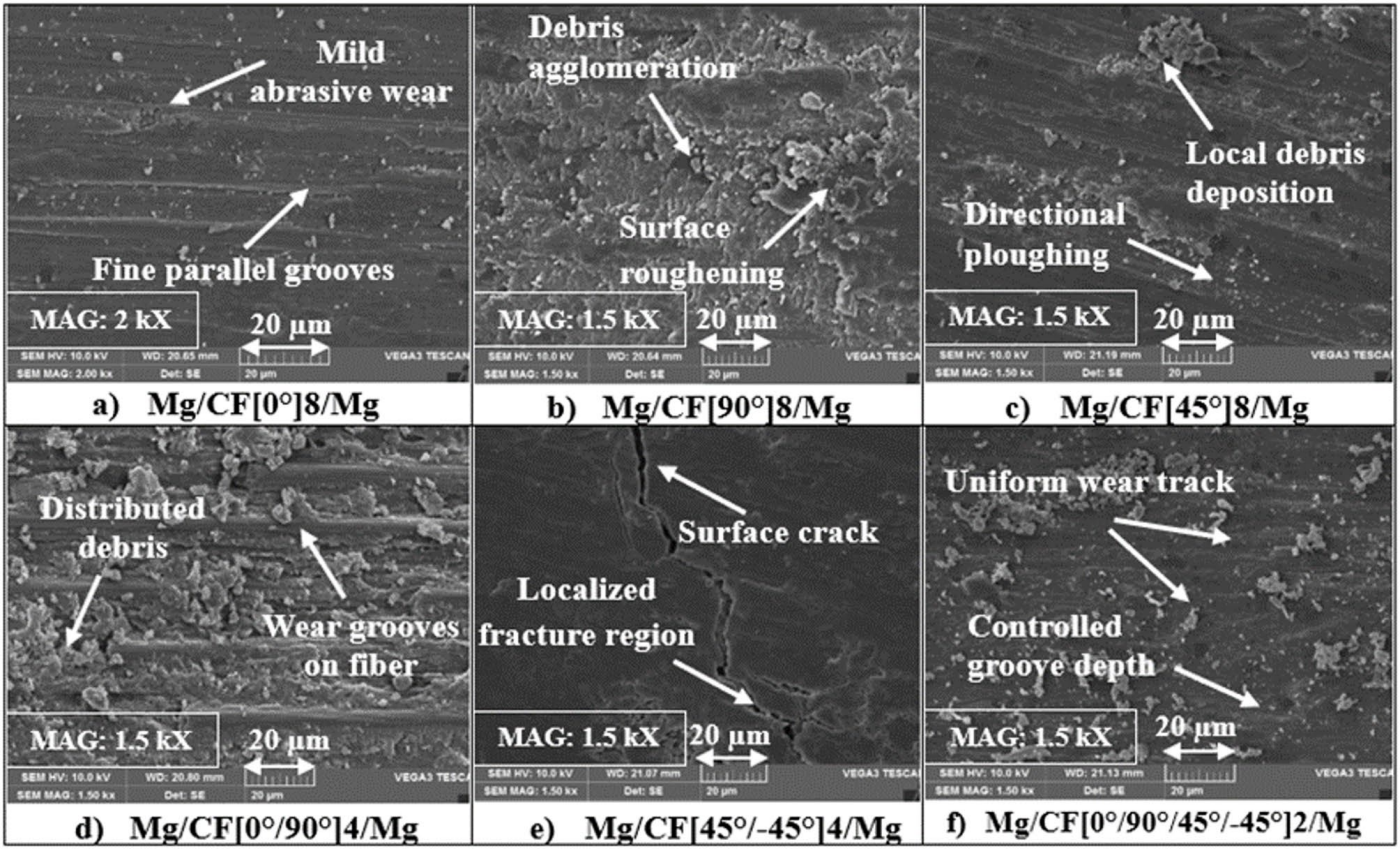
SEM micrographs of worn CFRP surface morphology along the wear track for Mg-CF sandwich composites with different stacking configurations.

The SEM micrographs of the worn CFRP surfaces (Fig. 19) reveal distinct sliding-induced damage features depending on stacking configuration. The SEM analysis further shows that the dominant wear mechanisms across all configurations include abrasive micro-ploughing, matrix smearing, fibre exposure, debris formation, and localised cracking. The morphology of the worn surfaces exhibits a strong dependence on fibre orientation, which governs load transfer, interfacial interaction, and resistance to material removal during sliding.

In the Mg/CF[0°]_8_/Mg configuration, the worn surface is characterised by fine, shallow grooves aligned along the sliding direction, with average groove widths of approximately 2–4 μm and small debris particles (< 8 μm), indicating mild abrasive wear and stable sliding conditions. In contrast, the Mg/CF[90°]_8_/Mg laminate exhibits severe surface degradation, with irregular grooves ranging from 10 to 18 μm in width, debris agglomerates of approximately 15–30 μm, and localised cracks extending up to ~ 100 μm. These features reflect unstable wear behaviour and increased interfacial damage due to unfavourable fibre orientation. The Mg/CF[45°]_8_/Mg configuration shows moderate wear characteristics, with directional ploughing grooves of approximately 5–9 μm and debris clusters in the range of 10–18 μm, indicating intermediate tribological performance. The Mg/CF[0°/90°]_4_/Mg laminate exhibits comparatively uniform surface morphology, with groove widths of approximately 4–7 μm and evenly distributed debris (~ 8–15 μm), suggesting improved wear stability due to balanced fibre orientation.

More severe surface damage is observed in the Mg/CF[45°/−45°]_4_/Mg configuration, where pronounced ploughing grooves (~ 8–14 μm), fibre pull-out regions with lengths of approximately 20–35 μm, and surface cracks extending up to ~ 80–120 μm are evident. These features indicate significant stress concentration and matrix-dominated failure. In contrast, the multidirectional Mg/CF[0°/90°/45°/−45°]_2_/Mg laminate demonstrates a relatively smooth and uniform wear track, with controlled groove widths of approximately 3–6 μm, fine debris particles (< 10 μm), and minimal fibre pull-out (< 15 μm), indicating enhanced wear resistance and stable sliding behaviour.

Overall, the worn surface morphology confirms that groove width, debris size, crack formation, and fibre pull-out are strongly influenced by fibre architecture. Multidirectional laminates exhibit reduced surface damage and improved tribological stability due to effective stress redistribution and enhanced interfacial bonding, whereas unidirectional and angle-ply configurations show increased susceptibility to severe wear mechanisms. These observations are in strong agreement with the measured wear rate, frictional behaviour, and surface roughness results, thereby reinforcing the critical role of fibre orientation and stacking sequence in governing the tribological performance of Mg-CF sandwich composites. These morphological observations provide qualitative support for the wear mechanisms discussed in Sect. 5.1.1, establishing a clear correlation between surface damage features and tribological behaviour.

### Microhardness characterization

Vickers microhardness measurements were performed to evaluate the resistance to localised plastic deformation in both the magnesium face sheets and the composite core cross-section of the Mg-CF sandwich laminates. Polished specimens were indented under a constant load of 100 gf (0.98067 N) with a dwell time of 15 s using a diamond pyramid indenter to ensure consistency across all stacking configurations. For each laminate, three independent indentations were made at uniformly spaced locations to improve repeatability and minimise the influence of microstructural heterogeneity.

**Fig. 20:**
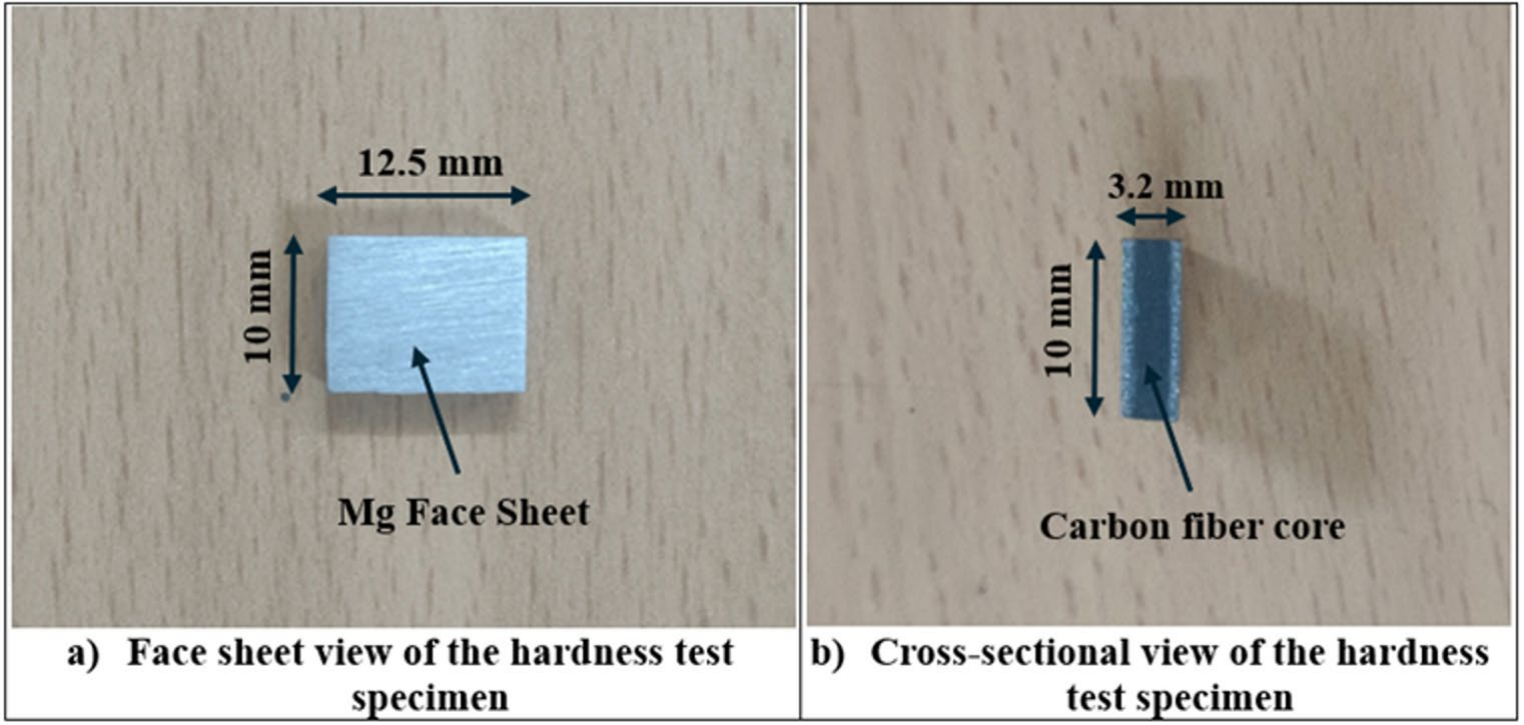
Geometry of the magnesium-carbon fiber sandwich composite specimen used for hardness testing.

All measurements were conducted under controlled laboratory conditions, and the reported values are expressed as mean ± standard deviation to ensure reliability and reproducibility. Figure 20 illustrates the magnesium face sheet dimensions and the cross-sectional configuration showing the CFRP core thickness considered for hardness evaluation. The lengths of the two diagonals of each indentation, $\:{d}_{1}$and $\:{d}_{2}$, were measured using the integrated optical microscope of the hardness tester. The mean diagonal length was calculated as:

13 $$\:d=\frac{{d}_{1}+{d}_{2}}{2}$$

Were d_1_ and d_2_ are the lengths of two diagonals.

The Vickers hardness number (HV) was then determined using the standard relation:

14 $$\:HV=\frac{1.8544{\hspace{0.17em}}P}{{d}^{2}}$$

where * P* = Applied load (N).

* d* = Average indentation diagonal length (in mm) = d (µm) × 10^− 3^.

**Table 9:** Indentation diagonal measurements and corresponding Vickers hardness (HV) values on each sample face sheet of the Mg-CF sandwich composite.

Configuration	Test	d₁ (µm)	d₂ (µm)	d (µm)	HV
Mg/CF[0°]_8_/Mg	1	51.12	52.5	51.81	69
	2	50.87	52	51.435	70
	3	50.5	52.87	51.685	69.4
Mg/CF[90°]_8_/Mg	1	52	51	51.5	69.9
	2	51.87	51.5	51.685	69.4
	3	51.5	51	51.25	70.6
Mg/CF[45°]_8_/Mg	1	50.87	51.25	51.06	71.1
	2	51.25	50.5	50.875	71.6
	3	51.87	51	51.435	70
Mg/CF[0°/90°]_4_/Mg	1	50.5	50.5	50.5	72.7
	2	50	49.5	49.75	74.9
	3	49.75	51.25	50.5	72.7
Mg/CF[45°/−45°]_4_/Mg	1	51.37	50.62	50.995	71.2
	2	51	48.37	49.685	75.1
	3	49.75	50.75	50.25	73.4
Mg/CF[0°/90°/45°/−45°]_2_/Mg	1	49.75	50.5	50.125	73.8
	2	50.25	50.25	50.25	73.4
	3	50.50	50.62	50.56	72.5

**Fig. 21:**
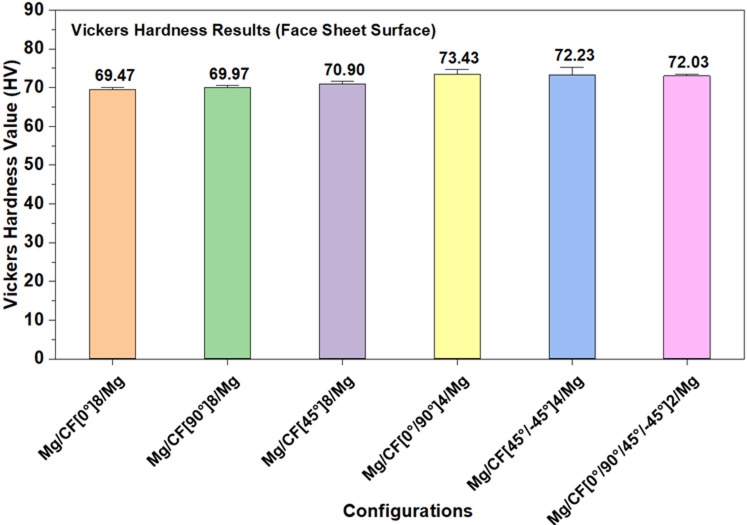
Vickers hardness of Mg-CF sandwich composite configurations measured at the face sheet surface.

Table 9 presents the measured indentation diagonals ($\:{d}_{1}$, $\:{d}_{2}$), the calculated average diagonal length (* d*), and the corresponding Vickers hardness values for each Mg-CF sandwich composite face sheet, providing the experimental basis for hardness evaluation. The average Vickers hardness values for the magnesium face sheets are summarised in Table 10 and range from 69.47 to 73.43 HV, indicating consistent metallurgical quality across all configurations. The corresponding hardness values are illustrated in Fig. 21.

**Table 10:** Average Vickers microhardness values (HV) and standard deviation for each sample face sheet of the Mg-CF sandwich composite.

Configuration	HV₁	HV₂	HV₃	Average HV	Std. Dev
Mg/CF[0°]_8_/Mg	69	70	69.4	69.47	± 0.50
Mg/CF[90°]_8_/Mg	69.9	69.4	70.6	69.97	± 0.60
Mg/CF[45°]_8_/Mg	71.1	71.6	70	70.90	± 0.82
Mg/CF[0°/90°]_4_/Mg	72.7	74.9	72.7	73.43	± 1.27
Mg/CF[45°/−45°]_4_/Mg	71.2	75.1	73.4	73.23	± 1.96
Mg/CF[0°/90°/45°/−45°]_2_/Mg	73.8	73.4	72.5	73.03	± 0.47

**Fig. 22:**
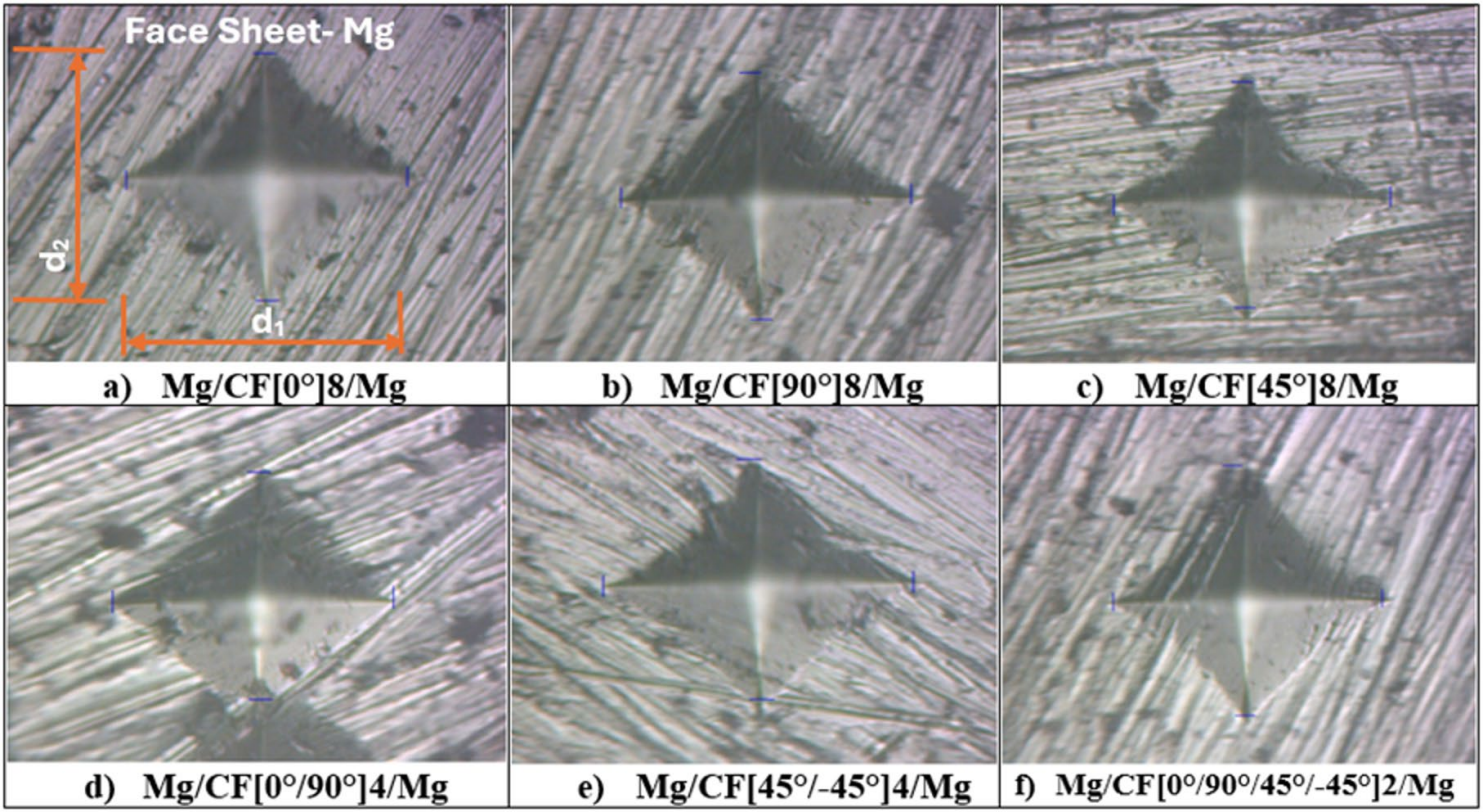
Optical micrographs of Vickers microhardness indentations on the magnesium face sheet of Mg-CF sandwich composites for different stacking configurations.

Among the investigated laminates, multidirectional and bidirectional stacking configurations exhibit comparatively higher face sheet hardness than unidirectional laminates. This enhancement is attributed to improved constraint effects and more efficient load transfer from the composite core to the magnesium face sheet during indentation. In contrast, unidirectional laminates show slightly lower hardness due to anisotropic subsurface support, which leads to increased localised plastic deformation beneath the indenter. The low standard deviation values confirm the repeatability of the measurements and the consistent metallurgical quality of the magnesium face sheets. The optical micrographs shown in Fig. 22 display well-defined diamond-shaped Vickers impressions with clearly distinguishable diagonals, confirming proper surface preparation and controlled loading conditions. Configurations with higher hardness exhibit smaller indentation diagonals, reflecting greater resistance to localised plastic deformation, whereas unidirectional laminates show relatively larger impressions consistent with their lower hardness. No evidence of cracking, edge chipping, or indentation-induced delamination is observed around the impressions, indicating good metallurgical integrity of the AZ31 magnesium face sheets and effective structural support from the composite core.

**Fig. 23:**
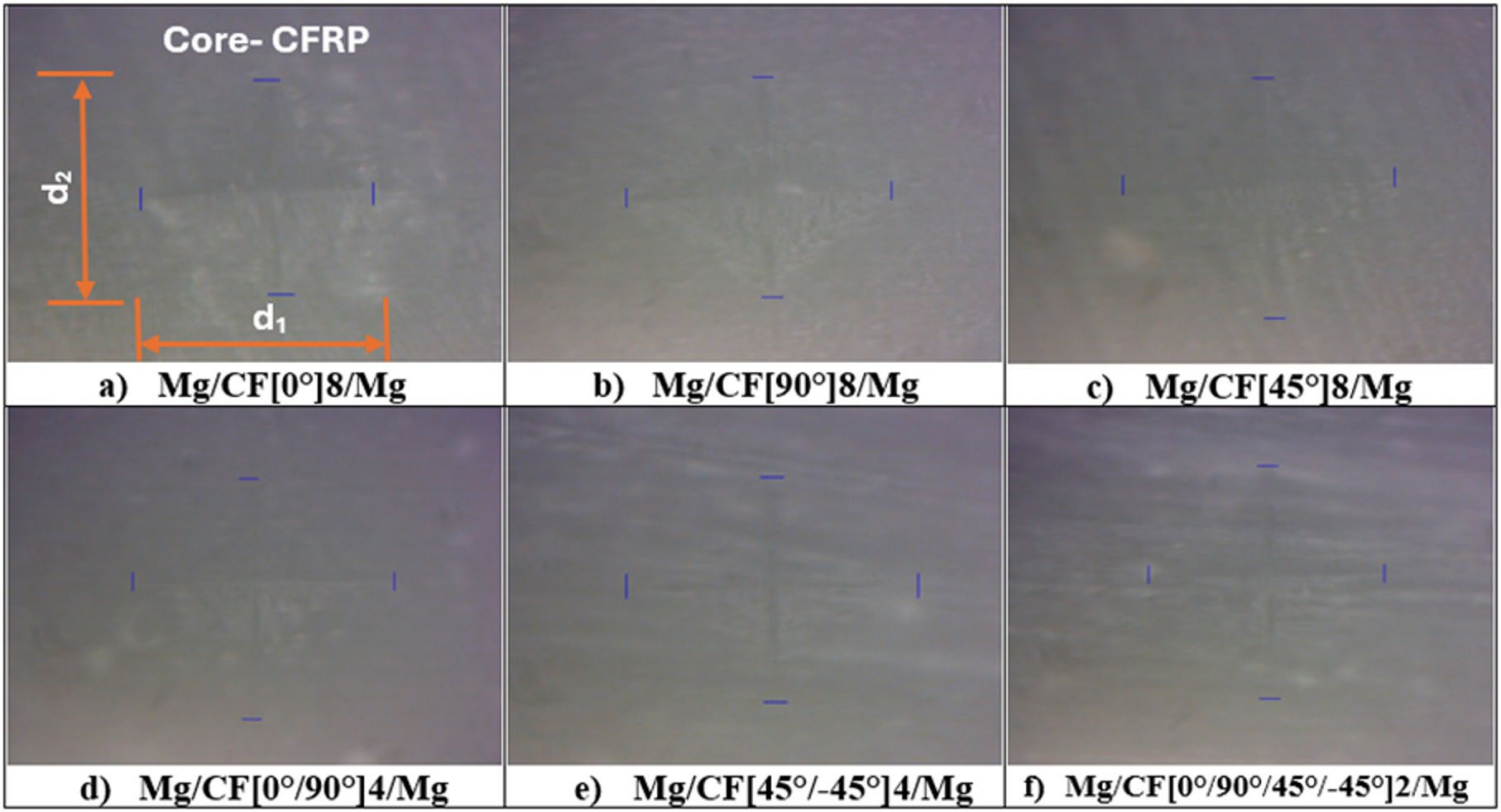
Optical micrographs of Vickers microhardness indentations on the core cross-section of Mg-CF sandwich composites for different stacking configurations.

Figure 23 presents the optical micrographs of Vickers indentations formed on the composite core cross-section of the Mg-CF sandwich laminates. Compared to the face sheet, the core indentations appear less sharply defined due to the heterogeneous nature of the carbon fiber-reinforced polymer (CFRP) structure and the localised interaction between fibre and matrix during indentation. Noticeable differences in indentation size and morphology are observed among the configurations, consistent with variations in core hardness. The multidirectional Mg/CF[0°/90°/45°/−45°]_2_/Mg laminate exhibits relatively smaller and more confined impressions, indicating higher resistance to localised deformation. Similarly, the Mg/CF[0°/90°]_4_/Mg configuration shows constrained indentation geometry, reflecting improved load distribution within the core, whereas the unidirectional Mg/CF[0°]_8_/Mg laminate exhibits larger deformation zones. The indentation boundaries remain continuous without visible macro-cracking or delamination, indicating satisfactory interfacial integrity under the applied load.

Table 11 presents the measured indentation diagonal lengths ($\:{d}_{1}$, $\:{d}_{2}$), and the corresponding Vickers hardness values obtained from the cross-sectional core region of the composites, while Table 12 summarises the average core hardness values along with their corresponding standard deviations for all configurations. The core hardness values exhibit a wider variation compared to the face sheets, ranging from 29.83 to 47.27 HV, highlighting the strong dependence of core mechanical behaviour on fibre orientation, stacking sequence, and interfacial characteristics. Among the configurations, the multidirectional Mg/CF[0°/90°/45°/−45°]_2_/Mg laminate demonstrates the highest core hardness (47.27 HV), followed by Mg/CF[0°/90°]_4_/Mg laminate (42.47 HV). The improved hardness in these configurations is attributed to multidirectional reinforcement, which enhances fibre interlocking and enables more efficient stress redistribution within the composite core during indentation.

**Table 11:** Indentation diagonal measurements and corresponding Vickers hardness (HV) Values on each sample core sheet cross-section of the Mg-CF Sandwich Composite.

Configuration	Test	d₁ (µm)	d₂ (µm)	d (µm)	HV
Mg/CF[0°]_8_/Mg	1	76	81.87	78.935	29.8
	2	73.87	86.5	80.185	29
	3	72.75	82.87	77.81	30.7
Mg/CF[90°]_8_/Mg	1	72.62	78.25	75.435	32.6
	2	72.37	77.75	75.06	32.9
	3	72.75	76.12	74.435	33.4
Mg/CF[45°]_8_/Mg	1	69.12	79.62	74.37	33.6
	2	74.25	81	77.625	30.8
	3	74	80.25	77.125	31.2
Mg/CF[0°/90°]_4_/Mg	1	64.37	69.75	67.06	41.2
	2	64.75	67.37	66.06	42.5
	3	61.25	69.25	65.25	43.7
Mg/CF[45°/−45°]_4_/Mg	1	68.75	78.12	73.435	34.5
	2	68.12	80.25	74.185	33.9
	3	62.25	75.62	68.935	39.3
Mg/CF[0°/90°/45°/−45°]_2_/Mg	1	61	62.37	61.685	48.7
	2	62.25	65.37	63.81	45.5
	3	60.37	64.50	62.435	47.6

**Table 12:** Average vickers Microhardness Values (HV) and standard deviation for each sample core sheet cross-section of the Mg-CF sandwich composite.

Configuration	HV₁	HV₂	HV₃	Average HV	Std. Dev
Mg/CF[0°]_8_/Mg	29.8	29	30.7	29.83	±0.85
Mg/CF[90°]_8_/Mg	32.6	32.9	33.4	32.97	±0.40
Mg/CF[45°]_8_/Mg	33.6	30.8	31.2	31.87	±1.52
Mg/CF[0°/90°]_4_/Mg	41.2	42.5	43.7	42.47	±1.25
Mg/CF[45°/−45°]_4_/Mg	34.5	33.9	39.3	35.90	±3.00
Mg/CF[0°/90°/45°/−45°]_2_/Mg	48.7	45.5	47.6	47.27	±1.62

Conversely, the unidirectional laminates Mg/CF[0°]_8_/Mg (29.83 HV) and Mg/CF[90°]_8_/Mg (32.97 HV) exhibit lower hardness due to limited transverse constraint and orientation-dependent deformation behaviour. The Mg/CF[45°/−45°]_4_/Mg configuration shows moderate hardness (35.90 HV) with comparatively higher scatter (± 3.00 HV), indicating localised heterogeneity in fibre distribution and interfacial bonding.

The observed variation in core hardness is governed by fibre orientation-dependent load-transfer mechanisms. In multidirectional laminates, fibres aligned along multiple directions provide enhanced resistance to indentation-induced deformation by enabling effective stress redistribution within the CFRP core. In contrast, unidirectional laminates exhibit anisotropic load transfer, where the absence of transverse reinforcement limits resistance to indentation loads. Additionally, improved fibre-matrix interfacial interaction in multidirectional architectures enhances load sharing and contributes to higher resistance against localised deformation.

**Fig. 24:**
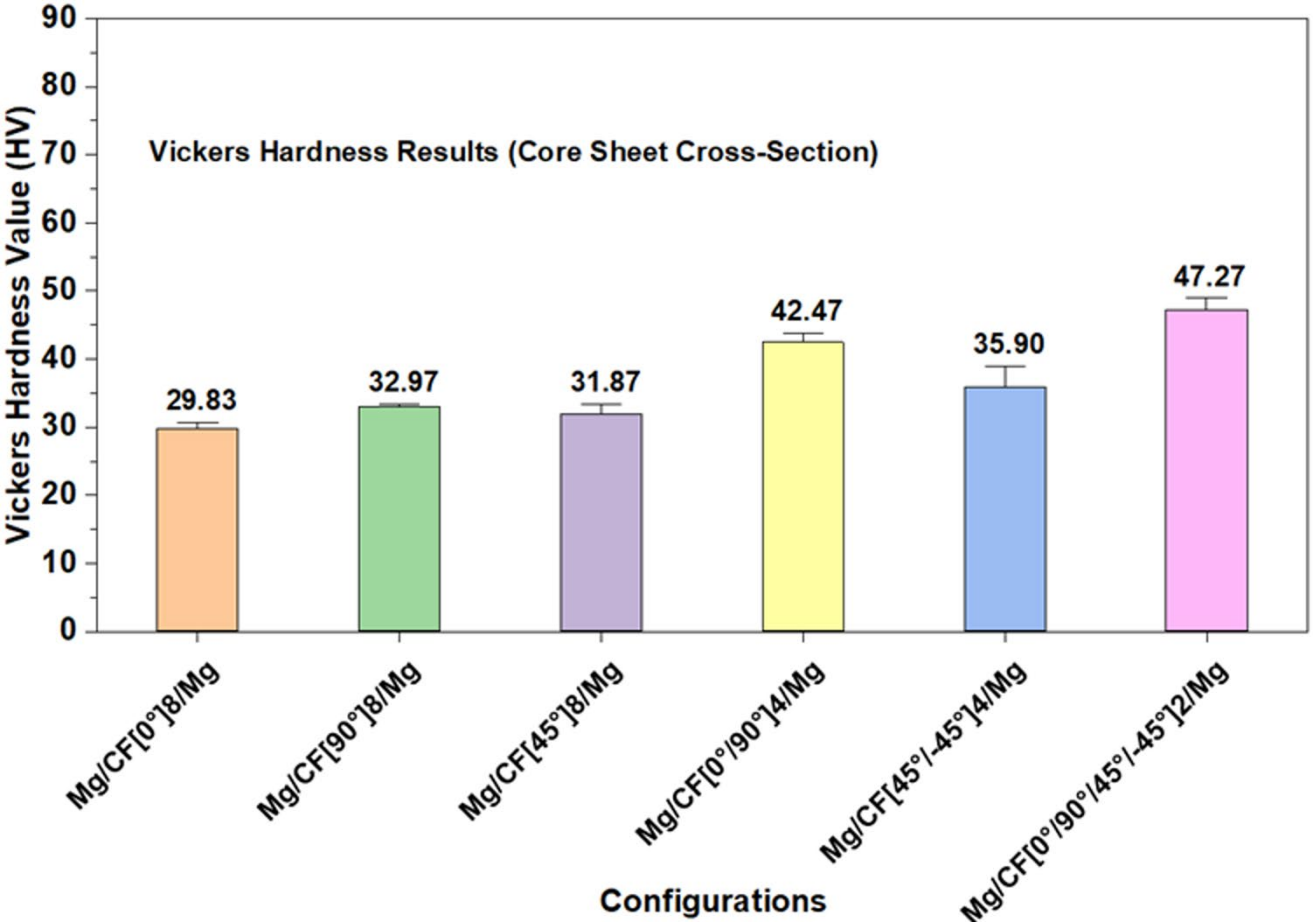
Vickers hardness of Mg-CF sandwich composite configurations measured at the core sheet cross-section.

The Vickers hardness values measured at the composite core cross-section for different stacking configurations are illustrated in Fig. 24. The results confirm that the mechanical performance of the core is strongly influenced by fibre orientation and stacking sequence, with multidirectional laminates demonstrating superior structural stability and load-sharing capability. A regression analysis between hardness and specific wear rate yielded a negative slope (y = − 2 × 10⁻⁵x + 0.0018) with a low coefficient of determination (R² = 0.2289), indicating a weak correlation. This suggests that wear behaviour is governed by multiple interacting factors rather than hardness alone. These findings are consistent with the tribological results, where configurations exhibiting higher core hardness also show lower wear rates and improved frictional stability, thereby reinforcing the critical role of fibre architecture in governing the overall performance of Mg-CF sandwich composites.

## Conclusions

This study systematically investigated the influence of fiber orientation and stacking sequence on the tribological behaviour, surface roughness evolution, and microhardness of magnesium-carbon fiber (Mg-CF) sandwich composites, establishing a clear structure-property relationship under dry sliding conditions. The findings establish that cross-sectional architecture plays a critical role in determining tribological performance, providing a novel perspective beyond conventional surface wear studies of Mg-CFRP composites.

The tribological performance of the Mg-CF sandwich composites was found to be strongly governed by fiber architecture. The multidirectional Mg/CF[0°/90°/45°/−45°]_2_/Mg laminate exhibited the lowest specific wear rate (0.86 × 10⁻³ mm³/N·m), whereas the unidirectional Mg/CF[90°]_8_/Mg configuration showed the highest wear rate (1.54 × 10⁻³ mm³/N·m), corresponding to an approximate 44% reduction in wear. The coefficient of friction varied between 0.145 and 0.363, with multidirectional stacking reducing friction by nearly 60% compared to the 90° unidirectional configuration, indicating improved sliding stability and load-sharing capability.

Surface roughness analysis further confirmed orientation-dependent wear behaviour, with roughness increment (ΔRa) ranging from 0.42 μm to 1.21 μm. The multidirectional laminate showed the lowest ΔRa (0.42 μm), while the angle-ply Mg/CF[45°/−45°]_4_/Mg exhibited the highest increase (1.21 μm), demonstrating greater susceptibility to ploughing and surface disruption in certain architectures.

Microhardness evaluation revealed that the magnesium face sheets maintained relatively uniform hardness (69.47–73.43 HV), indicating consistent metallurgical integrity independent of stacking configuration. In contrast, the composite core exhibited significant variation, ranging from 29.83 HV (Mg/CF[0°]_8_/Mg) to 47.27 HV (Mg/CF[0°/90°/45°/−45°]_2_/Mg), representing nearly 58% improvement in hardness for multidirectional laminates. This enhancement is attributed to improved fibre interlocking and more effective stress redistribution within the core.

SEM analysis confirmed that abrasive micro-ploughing and matrix smearing are the dominant wear mechanisms. Multidirectional laminates exhibited more uniform wear tracks, reduced debris accumulation, and minimal interfacial damage, whereas unidirectional configurations showed pronounced groove formation, fibre pull-out, and localised damage. These observations are consistent with the measured wear, roughness, and hardness results.

Overall, the results establish a strong interdependence between fibre architecture, mechanical response, and tribological behaviour. Multidirectional stacking (Mg/CF[0°/90°/45°/−45°]_2_/Mg) significantly enhances wear resistance, frictional stability, and structural integrity, making it a favourable configuration for applications involving sustained sliding contact.

This study establishes a comprehensive framework linking fibre orientation, load-transfer mechanisms, interfacial stability, and damage evolution in Mg-CF sandwich composites. The results provide new insights into the role of multidirectional reinforcement in enhancing tribological durability, which remains relatively underexplored in magnesium-based sandwich systems. The study further demonstrates the potential of optimised Mg-CF sandwich structures for lightweight aerospace and structural applications, including sliding interfaces, bearing components, and load-bearing panels. Tailoring fibre architecture significantly improves wear resistance and frictional stability, thereby contributing to extended service life, reduced maintenance requirements, and enhanced reliability under repeated sliding conditions.

The present study is limited to tribological evaluation under a single applied load condition during pin-on-disc testing, which restricts the analysis of wear mechanism transitions under varying loading environments. Future work will focus on investigating the effect of different normal loads and sliding conditions to develop a more comprehensive understanding of the tribological behaviour of Mg-CF sandwich composites.

## Future scope

Future research should extend the present tribological investigation by evaluating Mg-CF sandwich composites under varying normal loads, sliding velocities, and environmental conditions such as elevated temperature, humidity, and corrosive atmospheres to better represent realistic service environments. In particular, high temperature tribological studies are essential to understand thermally induced softening, oxidation behaviour, and interfacial stability during sliding. Long-term durability assessment through fatigue-wear and fretting wear studies is necessary to capture cyclic loading and vibration-induced damage commonly encountered in aerospace applications. In addition, depth-dependent nanoindentation across the Mg-CFRP interface can provide detailed insight into localised mechanical gradients and interfacial load-transfer behaviour.

Further performance improvements may be achieved through advanced surface engineering techniques, including plasma treatment, protective ceramic or hybrid coatings, and nano-reinforcement strategies to enhance wear and corrosion resistance. The development of validated finite element models incorporating anisotropic material behaviour and interfacial characteristics would enable more accurate prediction of stress distribution and wear-induced deformation mechanisms. Coupled impact-wear studies may also provide deeper insight into damage evolution under complex loading conditions.

Finally, future work should focus on component-level validation through the fabrication and testing of prototype structures to assess scalability, manufacturability, and real-world tribological reliability of Mg-CF sandwich composites.

## Data Availability

The data supporting the findings of this study are available from the corresponding author upon reasonable request.

## References

[1] Simões, S. High-performance advanced composites in multifunctional material design: State of the art, challenges, and future directions. *Materials* **17**(23), 5997. 10.3390/ma17235997 (2024). 39685433 10.3390/ma17235997 PMC11643371

[2] Kausar, A., Ahmad, I., Rakha, S. A., Eisa, M. H. & Diallo, A. State-of-the-art of sandwich composite structures: Manufacturing—to—high performance applications. *J. Compos. Sci.* **7**(3), 102. 10.3390/jcs7030102 (2023).

[3] Jhamb, S., Matai, J., Marwaha, J., Goyal, A. & Pandey, A. A comprehensive analysis on magnesium-based alloys and metal matrix composites for their in-vitro biocompatibility. *Adv. Mater. Process. Technol.* **9**(3), 1249–1282. 10.1080/2374068X.2022.2113521 (2023).

[4] Kumar, S. D., Arulmurugan, B., Muthukumaran, N. & Babu, S. R. A Comprehensive Survey on Friction-Based Processing of AZ Series Magnesium Alloys. Advances in Processing of Lightweight Metal Alloys and Composites: Microstructural Characterization and Property Correlation, 231–248. (2022). 10.1007/978-981-19-7146-4_13

[5] Lv, G. et al. Microstructure, mechanical and wear properties of short carbon fiber-reinforced AM50 magnesium matrix composite. *Int. J. Metacast.* **18**(4), 3028–3046. 10.1007/s40962-023-01220-5 (2024).

[6] Settu, S. S., Midhunchakkaravarthy, D., Malkapuram, M. E. A., Ramalingam, R. & R., & Impact response and residual performance of hybrid graded sandwich panels for UAV structural applications. *J. Sandw. Struct. Mater.* 10996362261444130. 10.1177/10996362261444130 (2026).

[7] Sahu, S. K., Sreekanth, P. R. & Reddy, S. K. A brief review on advanced sandwich structures with customized design core and composite face sheet. *Polymers* **14**(20), 4267. 10.3390/polym14204267 (2022). 36297845 10.3390/polym14204267 PMC9608463

[8] Yarlagaddaa, J., Malkapuram, R. & Balamurugan, K. Machining studies on various ply orientations of glass fiber composite. In Advances in Industrial Automation and Smart Manufacturing: Select Proceedings of ICAIASM 2019 (pp. 753–769). Singapore: Springer Singapore. (2020). 10.1007/978-981-15-4739-3_67

[9] Jang, W. C. & Roh, H. D. Sandwich composites manufacturing: A review of materials, methods, applications and challenges. *Int. J. Precis. Eng. Manuf.* **26**(8), 2093–2109. 10.1007/s12541-025-01285-8 (2025).

[10] Lei, L. et al. Effect of foam copper interlayer on the mechanical properties and fretting wear of sandwich clinched joints. *J. Mater. Process. Technol.* **274**, 116285. 10.1016/j.jmatprotec.2019.116285 (2019).

[11] Benincá, F. P. et al. Evolution of morphology, microstructure and hardness of bodies and debris during sliding wear of carbon steels in a closed tribosystem. *Wear* **523**, 204809. 10.1016/j.wear.2023.204809 (2023).

[12] Pintaude, G. Hardness as an indicator of material strength: a critical review. *Crit. Rev. Solid State Mater. Sci.* **48** (5), 623–641. 10.1080/10408436.2022.2085659 (2023).

[13] Sathiyamurthy, S., Saravanakumar, S. & Vinoth, V. Enhancing tribological performance of hybrid fiber-reinforced composites through machine learning and response surface methodology. *J. Reinf. Plast. Compos.* **44** (21–22), 2306–2324. 10.1177/07316844241256421 (2025).

[14] Yang, Z., Wang, Z., Wang, J., Li, Z. & Liu, X. Tribological properties of SiCp/A356 composites against semimetallic materials under dry and wet conditions. *J. Mater. Eng. Perform.* **30** (6), 4148–4161. 10.1007/s11665-021-05722-3 (2021).

[15] Rustamov, I., Xiang, L., Xia, Y. & Peng, W. Tribological and mechanical endowments of polyoxymethylene by liquid-phase exfoliated graphene nanofiller. *Polym. Int.* **74** (3), 231–245. 10.1002/pi.6712 (2025).

[16] Sagar, P., Khanna, S. K., Vignjevic, R. & Handa, A. Synergistic effect of hybrid reinforcement on magnesium-based composites for enriching mechanical and tribological characteristics. *Proc. Institution Mech. Eng. Part. E: J. Process. Mech. Eng.* **239** (5), 2915–2928. 10.1177/09544089231221683 (2025).

[17] Duan, J. et al. Tribological behavior and applications of carbon fiber reinforced ceramic composites as high-performance frictional materials. *Ceram. Int.* **47**(14), 19271–19281. 10.1016/j.ceramint.2021.02.187 (2021).

[18] Ataya, S. et al. Wear characteristics of Mg alloy AZ91 reinforced with oriented short carbon fibers. *Materials* **15**(14), 4841. 10.3390/ma15144841 (2022). 35888308 10.3390/ma15144841 PMC9324677

[19] Zhang, H. et al. Wear resistance mechanisms of fibre-reinforced lubrication on textured surfaces. *J. Manuf. Process.* **149**, 647–663. 10.1016/j.jmapro.2025.05.056 (2025).

[20] Jalilvand, M. M. & Mazaheri, Y. Effect of mono and hybrid ceramic reinforcement particles on the tribological behavior of the AZ31 matrix surface composites developed by friction stir processing. *Ceram. Int.* **46** (12), 20345–20356. 10.1016/j.ceramint.2020.05.123 (2020).

[21] Kumar, T. S., Shalini, S., Petrů, J., Kirubavathi, G. & Kalita, K. Machine learning-based prediction of wear behaviour of AZ31 hybrid composites reinforced with NbC and ZrC. *Sci. Rep.* **15**(1), 42056. 10.1038/s41598-025-26093-y (2025). 41298744 10.1038/s41598-025-26093-y PMC12657862

[22] Milosevic, M., Valášek, P. & Ruggiero, A. Tribology of natural fibers composite materials: an overview. *Lubricants* **8** (4), 42. 10.3390/lubricants8040042 (2020).

[23] Wang, Y. & Zhang, J. A review of the friction and wear behavior of particle-reinforced aluminum matrix composites. *Lubricants* **11**(8), 317. 10.3390/lubricants11080317 (2023).

[24] Zheng, H. et al. 3D printing continuous fiber reinforced polymers: A review of material selection, process, and mechanics-function integration for targeted applications. *Polymers* **17**(12), 1601. 10.3390/polym17121601 (2025). 40574129 10.3390/polym17121601 PMC12196871

[25] Lv, X. et al. Tribological anisotropy of PEEK composites filled with highly oriented carbon fibers manufactured by fused deposition modeling. *Polym. Compos.* **45**(3), 2656–2669. 10.1002/pc.27946 (2024).

[26] Kumar, P. & Srivastava, V. K. Reciprocating sliding tribology of ceramic fiber composites with variation of laminate orientation and surface conformity. *Ceram. Int.* **44** (5), 5365–5370. 10.1016/j.ceramint.2017.12.160 (2018).

[27] Fidan, S., Ürgün, S., Özsoy, M. İ, Bora, M. Ö. & Güleç, E. Tribological response of glass fiber/polyester composites after pressurized water-immersion aging assessed by reciprocating and ball-on-disc wear testing. *Polymers* **17**(18), 2503. 10.3390/polym17182503 (2025). 41012267 10.3390/polym17182503 PMC12473698

[28] Demiral, M. Strength in adhesion: A multi-mechanics review covering tensile, shear, fracture, fatigue, creep, and impact behavior of polymer bonding in composites. *Polymers* **17** (19), 2600. 10.3390/polym17192600 (2025). 41096246 10.3390/polym17192600 PMC12526568

[29] Aydın, F. Tribological aspects of magnesium matrix composites: A review of recent experimental studies. *Tribol. Mater. Surf. Interfaces.* **17**(4), 363–396. 10.1080/17515831.2023.2246809 (2023).

[30] Banerjee, S., Sahoo, P. & Davim, J. P. Tribological characterisation of magnesium matrix nanocomposites: A review. *Adv. Mech. Eng.* **13**(4), 16878140211009025. 10.1177/16878140211009025 (2021).

[31] Essam, M. A., Faragallah, M. M., Abdeltawab, N. M. & Ali, M. Tribological characteristics of composite brake pads under variable load and speed. *Sci. Rep.*10.1038/s41598-025-33326-7 (2026). 41526415 10.1038/s41598-025-33326-7 PMC12800048

[32] Niwas, R. & Kumar, V. Exploring hardness and tribological performance of rutile/silver reinforced magnesium composites fabricated by friction stir processing. *Ceram. Int.*10.1016/j.ceramint.2025.06.431 (2025).

[33] Nisar, L., Maqbool, A., Khan, N. Z., Gull, A. & Siddiquee, A. N. Enhancing ductility, strength, and hardness of WE43 Mg alloy-based metal matrix composite fabricated via friction stir processing: Effect of hybrid reinforcement and volume percentage. *Mater. Chem. Phys.* **323**, 129620. 10.1016/j.matchemphys.2024.12962 (2024).

[34] Ammisetti, D. K., Sai Sarath, K. & Kruthiventi, S. H. A review on mechanical and wear characteristics of magnesium metal matrix composites. *J. Tribol.* **147**(2), 020801. 10.1115/1.4066416 (2025).

[35] Patel, V. K., Rana, H. & Patel, D. K. Study on AZ31 magnesium alloy-based surface composite through friction stir processing: A review. *Weld. Int.* **39**(4), 280–296. 10.1080/09507116.2025.2458043 (2025).

[36] Rajak, D. K., Wagh, P. H. & Linul, E. Manufacturing technologies of carbon/glass fiber-reinforced polymer composites and their properties: A review. *Polymers* **13**(21), 3721. 10.3390/polym13213721 (2021). 34771276 10.3390/polym13213721 PMC8588351

[37] Anirudh, S., Prabha, K. A., Murthy, S. B. & Rao, B. C. Enhancement of mechanical properties of matrix by functionalized multi-wall carbon nanotubes (MWCNT) in composite material. *Mater. Today Proc.* **92**, 1186–1192. 10.1016/j.matpr.2023.05.30 (2023).

[38] Zhang, W. et al. Microstructure evolution and mechanical properties of AZ31 magnesium alloy sheets prepared by low-speed extrusion with different temperature. *Crystals* **10**(8), 644. 10.3390/cryst10080644 (2020).

[39] Annadorai, M. E. & Ramakrishna, M. Mechanical and thermal performance of magnesium carbon fiber sandwich composites with variable fiber orientations for aerospace structures. *Sci. Rep.*10.1038/s41598-026-38567-8 (2026). 41654620 10.1038/s41598-026-38567-8 PMC12946178

[40] Srivastava, A. K., Saxena, A. & Dixit, A. R. Investigation on the thermal behaviour of AZ31B/waste eggshell surface composites produced by friction stir processing. *Compos. Commun.* **28**, 100912. 10.1016/j.coco.2021.100912 (2021).

[41] Asmawi, N., Kuan, T. M. & Siddiqui, V. U. Manufacturing of fiber polymer composite materials. In Aerospace Materials (pp. 333–347). Elsevier. (2025). 10.1016/B978-0-443-22118-7.00014-2

[42] Dackweiler, M., Hagemann, L., Coutandin, S. & Fleischer, J. Experimental investigation of frictional behavior in a filament winding process for joining fiber-reinforced profiles. *Compos. Struct.* **229**, 111436. 10.1016/j.compstruct.2019.111436 (2019).

[43] Yarlagaddaa, J. & Malkapuram, R. Influence of MWCNTs on the mechanical properties of continuous carbon epoxy composites. *Rev. Compos. Mater. Av.*10.18280/rcma.300106 (2020).

[44] Annadorai, M. E., Ramakrishna, M. & Jyothi, Y. Effect on mechanical properties of CFRP composites in different fiber orientations. *Interactions* **245**(1), 198. 10.1007/s10751-024-02035-4 (2024).

[45] Selvam, R., Arunkumar, N. & Karunamoorthy, L. An investigation on machining characteristics in abrasive water jet machining of hybrid laminated composites with SiC nano particles. *Mater. Today: Proc.* **39**, 1701–1709. 10.1016/j.matpr.2020.06.193 (2021).

[46] Hei, D., Zheng, M., Wang, X., Dong, X. & Zheng, Y. Wear behavior of the Al/Mg multilayered composite on the cross-sectional edge of the ARB-processed. *Trans. Indian Inst. Met.* **78**(7), 153. 10.1007/s12666-025-03647-2 (2025).

[47] Pushpanathan, D. P., Muthukumar, V. & Prem Kumar, S. Rajasekar, Investigations on the microstructure and microhardness of the friction stir processed AZ80 surface composites. In AIP Conference Proceedings (Vol. 2463, No. 1, p. 020059). AIP Publishing LLC. (2022)., May 10.1063/5.0080389

[48] Rooprai, R. S., Singh, V., Singh, J., Bansal, A. & Singla, A. K. Optimizing wear performance in vanadium-carbide-reinforced AISI316 composite claddings via wire arc additive manufacturing. *J. Mater. Eng. Perform.* **34**(23), 28374–28390. 10.1007/s11665-025-11312-4 (2025).

[49] Kaushik, J. et al. A review on application and optimization processes used for wear testing machine (pin on disc apparatus). *Mater. Today Proc.* **64**, 1440–1444. 10.1016/j.matpr.2022.04.735 (2022).

[50] Fernandes, M. et al. Synthesis of polymeric microcapsules filled with castor oil to enhance tribological properties in epoxy resin. *J. Compos. Mater.* **58**(25), 2739–2754. 10.1177/00219983241276932 (2024).

[51] Chu, H. et al. Vickers hardness distribution and prediction model of cement pastes corroded by sulfate under the coexistence of electric field and chloride. *Constr. Build. Mater.* **309**, 125119. 10.1016/j.conbuildmat.2021.125119 (2021).

[52] Husain, N. A. H., Feilzer, A. J., Kleverlaan, C. J., Abou-Ayash, S. & Özcan, M. Effect of hydrothermal aging on the microhardness of high-and low-viscosity conventional and additively manufactured polymers. *J. Prosthet. Dent.* **128**(4), 822-e1. 10.1016/j.prosdent.2022.08.022 (2022). 10.1016/j.prosdent.2022.08.022 36202632

[53] Yu, X. et al. Tensile properties, time-dependent deformation and damage mechanisms of polyester-carbon fiber helical auxetic yarn/polyurea composite coatings. *Mech. Time-Depend. Mater.* **29**(3), 69. 10.1007/s11043-025-09807-7 (2025).

[54] Shubham, S. K., Pandey, A. & Purohit, R. Study of tribological behavior using the Taguchi method and pin-on-disc machine along with mechanical properties of hybrid polymer composites. *Colloid Polym. Sci.* **303**(4), 563–577. 10.1007/s00396-025-05377-9 (2025).

[55] Kaushik, J. et al. A review on application and optimization processes used for wear testing machine (pin on disc apparatus). *Mater. Today Proc.* **64**, 1440–1444. 10.1016/j.matpr.2022.04.73 (2022).

[56] Jain, A. et al. Effect of reinforced weave patterns on the mechanical performance and wear resistance of wool-epoxy composites. *Mater. Res. Express* **13**(1), 015303. 10.1088/2053-1591/ae32e6 (2026).

[57] Rattan, N. et al. Himalayan Sheep Wool Reinforced Composite-A Novel Sustainable Material for Future. *Curr. Mater. Sci.*10.2174/0126661454330432241003090151 (2024).

